# Endoglin inhibitor TRC105 with or without bevacizumab for bevacizumab-refractory glioblastoma (ENDOT): a multicenter phase II trial

**DOI:** 10.1038/s43856-023-00347-0

**Published:** 2023-09-08

**Authors:** Manmeet S. Ahluwalia, Lisa R. Rogers, Rekha Chaudhary, Herbert Newton, Ahmad Ozair, Atulya A. Khosla, Andrew B. Nixon, Bonne J. Adams, Ben K. Seon, David M. Peereboom, Charles P. Theuer

**Affiliations:** 1 Rose and Ella Burkhardt Brain Tumor and Neuro-Oncology Center, Cleveland Clinic, Cleveland, OH USA; 2 Miami Cancer Institute, Baptist Health South Florida, Miami, FL USA; 3 Herbert Wertheim College of Medicine, Florida International University, Miami, FL USA; 4 Department of Neurosurgery, Henry Ford Health, Detroit, MI USA; 5 Division of Hematology & Oncology, University of Cincinnati, Cincinnati, OH USA; 6 Department of Neurology, Ohio State University, Columbus, OH USA; 7 Department of Neurology, University Hospitals Cleveland Medical Center, Cleveland, OH USA; 8 Bloomberg School of Public Health, Johns Hopkins University, Baltimore, MD USA; 9 Department of Internal Medicine, William Beaumont University Hospital, Royal Oak, MI USA; 10 Duke Cancer Institute, Duke University, Durham, NC USA; 11 TRACON Pharmaceuticals Inc., San Diego, CA USA; 12 Department of Immunology, Roswell Park Comprehensive Cancer Center, Buffalo, NY USA

**Keywords:** CNS cancer, Chemotherapy, CNS cancer, Cancer therapeutic resistance

## Abstract

**Background:**

Glioblastoma (GBM), the most lethal primary brain tumor, has limited treatment options upon recurrence after chemoradiation and bevacizumab. TRC105 (carotuximab), a chimeric anti-endoglin (CD105) antibody, inhibits angiogenesis and potentiates activity of VEGF inhibitor bevacizumab in preclinical models. This study sought to assess safety, pharmacokinetics, and efficacy of TRC105 for bevacizumab-refractory GBM.

**Methods:**

We conducted a pre-registered (NCT01564914), multicenter, open-label phase II clinical trial (ENDOT). We administered 10 mg/kg TRC105 monotherapy (first cohort) in adults with GBM and radiographic progression following radiation, temozolomide and bevacizumab therapy. Primary outcome was median time-to-progression (TTP), amended after first cohort’s enrollment to median overall survival (mOS). Secondary outcomes were objective response rate, safety and tolerability, and progression-free survival (PFS).

**Results:**

6 patients were enrolled in TRC105 monotherapy cohort. Median TTP and PFS of 5 evaluable patients receiving monotherapy was 1.4 months, in whom plasma VEGF-A levels were elevated post-therapy. Lack of response led to protocol amendment, and second cohort’s addition of bevacizumab+TRC105 with primary endpoint of mOS. 16 patients were enrolled in bevacizumab+TRC105 cohort. mOS of 15 evaluable patients was 5.7 (95%CI: 4.2–9.8) months. All 22 patients had measurable disease at baseline. Median PFS of 14 evaluable patients receiving bevacizumab+TRC105 was 1.8 months (95%CI 1.2–2.1). Serum TRC105 was measurable above target concentration of 25 ug/mL in all evaluable patients. Study medications were well-tolerated in both cohorts. Combined administration did not potentiate known toxicities of either medication, with cerebral hemorrhage not observed.

**Conclusions:**

Single-agent TRC105 lacks activity in bevacizumab-refractory GBM, possibly secondary to upregulated VEGF-A expression. Meaningful mOS in bevacizumab+TRC105 cohort warrants further trials to investigate efficacy of combination therapy.

## Plain language summary

Glioblastoma is an aggressive and lethal brain tumor, with patients typically expected to survive for 14 to 16 months after diagnosis. Nearly all patients experience tumor recurrence once conventional treatment strategies fail, after which a drug called bevacizumab is used. However, subsequent treatment options are extremely limited. We performed a clinical trial in which we investigated how safe and effective a new drug called TRC105 (carotuximab) is in patients who no longer respond to chemotherapy, radiotherapy or bevacizumab. We tested TRC105 both with and without bevacizumab, since TRC105 might enhance the activity of bevacizumab. We found that patients survived for an average of 5.7 months when given TRC105 and bevacizumab in combination. These findings suggest that further clinical trials are needed to confirm whether or not this combination therapy is a useful approach in patients with glioblastoma recurrence.

## Abstract (web summary)

Ahluwalia et al. report findings from a multicenter phase II clinical trial of TRC105, a chimeric IgG1 endoglin-directed antibody, given with and without bevacizumab, in bevacizumab-refractory glioblastoma. TRC105 administration resulted in increased circulating VEGF-A and combined with bevacizumab resulted in a median survival of 5.7 months.

## Introduction

Glioblastoma (GBM) is the most aggressive and the most lethal primary brain tumor worldwide, with patients typically expected to survive for 14 to 16 months after diagnosis. Recurrence within a few years is considered nearly inevitable and treatment options available upon recurrence after the standard of care remain highly limited, despite decades of drug discovery and clinical trials. Given the highly vascular architecture of GBM, targeting angiogenesis in these tumors has been a long-standing area of effort. Classic studies in gliomas had previously demonstrated the dependence of tumoral growth on production and maintenance of the fragile tumor-associated vasculature^1,2^. Given the relatively high expression of vascular endothelial growth factor (VEGF) by glioma cells, it has been known that blockage of VEGF signaling led to C6 glioma xenografts in immunodeficient mice having hindered tumoral growth, impaired microvascular maturation, and reduction in tumor-associated macrophages^3^. Bevacizumab, a humanized monoclonal antibody targeting VEGF, is approved by the US Food and Drug Administration (FDA) for use in several human malignancies, including colorectal cancer, cervical cancer, ovarian cancer, renal cell carcinoma, and non–small-cell lung cancer, in various combination of other systemic therapies^4^.

Bevacizumab, as one of the extremely few successful drugs for GBM, carries substantial utility not in the frontline setting, but in managing GBM recurrence after chemoradiation. However, extremely limited treatment strategies remain when both chemo-radiotherapy and bevacizumab have failed, with most treatments providing only transient disease control. The BRAIN study, a phase II randomized clinical trial (RCT), evaluated the role of bevacizumab, alone or in combination with irinotecan, in 167 patients with recurrent GBM^5^. For the single-agent and combination cohorts, progression-free survival (PFS) at six months (PFS-6) was 42.6% and 50.3%, objective response rate (ORR) was 28.2% and 37.8%, and median overall survival (OS) was 9.2 months and 8.7 months, respectively. In a study from the US National Cancer Institute (NCI), 48 patients with recurrent GBM received bevacizumab producing an ORR of 35%, PFS-6 of 29%, and median OS of 31 weeks^6^. Clinical benefit was evident, in the form of reduced cerebral edema, tapered steroid doses, and improvement in neurological function, in nearly half of the patients. The addition of irinotecan after bevacizumab failure did not provide additional benefit. The US Food and Drug Administration (FDA) approved bevacizumab in patients with recurrent GBM based on single-agent response rates^4^. However, the duration of effect and the long-term efficacy of bevacizumab are limited. To date, clinical trials, including the BELOB trial and EORTC study with bevacizumab in recurrent GBM, have shown an improvement of PFS without improvement in OS^7,8^. Furthermore, there is no effective therapy for patients with recurrent GBM after progression on bevacizumab. Various studies have reported PFS of 5–8 weeks and OS of 3–5 months, emphasizing the dismal prognosis of these patients and an urgent need for effective options in this setting of bevacizumab-refractory recurrent GBM^6,9–12^ thus, there remains a great need for more effective agents. We hypothesize that anti-angiogenic approaches directed against non-VEGF endothelial targets will help inhibit angiogenesis, even after the GBM has developed resistance to therapies directed against VEGF and its receptors.

Potentiation of bevacizumab’s inhibition of the VEGF pathway may represent a potential strategy in rGBM. Endoglin (CD105) is a homodimeric TGF-β coreceptor expressed on proliferating vascular endothelium in solid tumors^13^. Endoglin is selectively expressed at high density on proliferating endothelial cells and is up-regulated by hypoxia through the induction of hypoxia-inducible factor-1-α (HIF-1-α)^14^. Endoglin expression is also up-regulated on tumor endothelial cells in response to inhibitors of the VEGF pathway and allows continued tumor growth^15,16^. Loss of endoglin expression reverses resistance to large and small molecule inhibitors of the VEGF pathway^17^. TRC105, also known as carotuximab, (TRACON Pharmaceuticals, Inc., San Diego, CA, USA) is a chimeric IgG1 antibody that binds human endoglin with high avidity, induces antibody-dependent cellular cytotoxicity (ADCC) and apoptosis of human vascular endothelial cells (HUVECs) and endoglin-positive tumor cells, and inhibits angiogenesis in response to VEGF and fibroblast growth factor. TRC105 potentiates bevacizumab in preclinical models of angiogenesis. It has been combined safely with bevacizumab in solid tumor patients^18^.

Therefore, this study sought to assess the safety, tolerability, pharmacokinetics, and antitumor activity of TRC105 when given concurrently with bevacizumab to GBM patients refractory to chemoradiation and bevacizumab through an open-label multicenter phase II clinical trial (ENDOT). The trial’s results, discussed below, indicate that single-agent TRC105 lacks activity for bevacizumab-refractory GBM, possibly secondary to upregulation of VEGF-A expression following TRC105 administration. However, the TRC105 plus bevacizumab combination therapy cohort had a median OS of 5.7 months. This promising data indicates further trials are warranted to further determine the efficacy of combination therapy.

## Methods

### Study design, ethics, and reporting

This work was initially planned as a multicenter, non-randomized, open-label, phase II exploratory clinical trial of single-agent TRC105 in patients with recurrent or progressive GBM. The study was prospectively registered on ClinicalTrials.Gov before accrual (NCT01564914) and conducted after approval by the Institutional Review Board (IRB) for all trial sites. The four centers in the US where the study was conducted and their IRB approvals were: (1) Cleveland Clinic, Cleveland Ohio – approval through Cleveland Clinic IRB (reference number 0000536); (2) University Hospitals, Case Western Reserve University, Ohio – approval through ‘C’ committee of the University Hospitals IRB (reference number IRB0000860) (2) the Ohio State University, Columbus, Ohio - approval through Western IRB (study number 1140617, WIRB protocol number 20130617); and (4) the University of Cincinnati, Cincinnati, Ohio. IRB approval at University Hospitals - approval through Western IRB (study number 1138648; WIRB protocol number 20130617).

Trial enrollment began on May 24, 2012, and ended on October 25, 2013. Trial registration may be viewed at https://www.clinicaltrials.gov/ct2/show/NCT01564914. Study procedures followed the Good Clinical Practice (GCP) guidelines, the Declaration of Helsinki, institutional standard operating procedures (SOPs), IRB policies, and all other applicable regulatory requirements. Appropriate procedures for obtaining informed consent from patients were in place. Due to a lack of activity in 6 patients treated with single-agent TRC105, the study was amended to an exploratory, non-randomized, open-label phase II clinical trial of TRC105, and a separate cohort added of TRC105 in combination with bevacizumab, with the primary endpoint changed from time to progression to median overall survival.

### Patient eligibility

Adult patients (aged ≥18 years) with histologically confirmed GBM, recurrent after prior external-beam fractionated radiotherapy and temozolomide chemotherapy, and with documented radiographic progression following bevacizumab were considered eligible. No more than three prior recurrences were allowed. Further eligibility criteria were standard, including Karnofsky performance status ≥70%, adequate organ function defined as an absolute neutrophil count ≥1.5  × 10^9^/L, hemoglobin ≥9 g/dL, platelets ≥100 × 10^9^/L, international normalized ratio (INR) ≤ 1.5, serum creatinine ≤1.5 times the upper limit of normal (ULN), serum total bilirubin ≤1.5 ULN, and aspartate transaminase (AST) and alanine transaminase (ALT) levels of ≤3.0 times ULN. Patients were required to have signed approved informed consent form and authorization regarding release of personal health information. Patients were required to have been at least 12 weeks since last radiation therapy, 2 weeks since last non-cytotoxic therapy, 3 weeks since completion of non-nitrosourea-containing-chemotherapy regimen, and at least 6 weeks since completion of nitrosourea-containing chemotherapy regimen. Patients were to have had no concurrent malignancy except (1) curatively treated basal or squamous cell carcinoma of the skin, (2) carcinoma in situ of the cervix or breast, or (3) adequately treated stage I or II cancer from which the patient was in complete remission. Patients were required to have been maintained on a stable or decreasing corticosteroid regimen from the time of their baseline scan until the start of treatment and/or for at least 5 days before starting treatment. Female patients of child-bearing potential were required to have a pregnancy test. A mini-mental state examination score of 15 or more was required.

Patients were excluded if they had received prior treatment with TRC105 or were unwilling to comply with the protocol. Further exclusion criteria, as per standard neuro-oncology trials, were state of pregnancy or breastfeeding in women, known sensitivity to recombinant antibodies or other study medications, active bleeding, pathological conditions with high-risk of bleeding, known diagnosis of HIV infection, cirrhosis or active hepatitis, impaired cardiac function or clinically significant cardiac diseases, evidence of active cardiovascular, cerebrovascular disease, or thromboembolic disease within six months of starting treatment, hemorrhage or unhealed surgical wounds within 30 days of study entry, uncontrolled hypertension, and current need for anticoagulation.

### Study intervention

All patients signed institutional review board (IRB)-approved informed consent form before undertaking study-related procedures. TRC105, manufactured in Chinese hamster ovary cells, was supplied as a phosphate-buffered saline solution in single-use glass vials for IV administration. Prior to infusion, the agent was diluted in normal saline and infused using an in-line 0.2-micron low-protein-binding filter. Pre-medications included acetaminophen, cetirizine (or a similar H1 receptor antagonist), famotidine (or similar H2 receptor antagonist), and dexamethasone 20 mg. Dexamethasone was tapered and discontinued as tolerated.

Single-agent TRC105 was dosed at 10 mg/kg weekly by intravenous infusion up to a maximum dose of 850 mg for women and 1000 mg for men of TRC105 on days 1, 8, 15, and 22 of each 28-day cycle, with the initial dose divided over two days by which 3 mg/kg was given on day 1, and 7 mg/kg was given on day 4.

When given with bevacizumab, TRC105 dosing began on cycle 1 day 8, and the initial 10 mg/kg dose was split into two doses, with 3 mg/kg administered on cycle 1 day 8 and 7 mg/kg administered on cycle 1 day 11. The entire weekly dose of TRC105 was then given on cycle 1 day 15 and weekly thereafter. The initial TRC105 infusion was given over four hours and the infusion time was reduced to a minimum of one hour, generally by the fifth weekly dose. Bevacizumab was administered as a 90 min infusion on cycle 1 day 1. Subsequent infusion times could be reduced in 30 min increments to a minimum infusion time of 30 min if tolerated. If the patient had received bevacizumab over 30 min in the past without the development of infusion reaction, the initial infusion on cycle 1 day 1, and all subsequent infusions could be given over 30 min.

Patients were eligible for continued treatment with bevacizumab and TRC105 until disease progression, as per modified Response Assessment in Neuro-Oncology (RANO) criteria, or unacceptable toxicity. Per protocol, patients were required to be withdrawn in case of medical necessity, investigator assessment, patient wishes, dose delay of 8 weeks or more, loss to follow-up or non-compliance, need for surgery, radiation or other anti-cancer therapy not specified in the protocol, or pregnancy.

### Pharmacokinetics and immunogenicity

Serum samples for assessment of TRC105 serum concentrations were collected prior to and following the third week of dosing (cycle 1 day 15), and TRC105 concentration was determined using a validated enzyme-linked immunosorbent assay (ELISA) with a limit of quantitation of 200 ng/mL. Serum for assessment of human antibody formation to TRC105 was collected before dosing, at the end of the study, and approximately four weeks following the last dose of TRC105, and titers were determined by validated ELISA-based tests.

### Study outcomes and assessments

The primary endpoint in the initially planned study was median time to progression (TTP) in patients with rGBM administered single-agent TRC105, defined as the time from consent to time of progression by RANO criteria. Later, in light of suboptimal response rates in the TRC105 monotherapy cohort, the study protocol was amended to include another cohort of recurrent GBM patients to be given TRC105 combined with bevacizumab, while the primary endpoint was designated as overall survival (OS) and TTP designated as a secondary endpoint. Thus, the final secondary endpoints were (1) safety and tolerability, as assessed by the NCI Common Terminology Criteria for Adverse Events (CTCAE) version 4.0 (the version in effect when the trial started accruing), (2) the overall response rate (ORR) as assessed by modified RANO criteria, (3) median TTP, (4) Progression-free survival (PFS) at six months (PFS-6), and (5) association between clinical outcome and soluble angiogenic biomarkers including but not limited to VEGF, PDGF, and TGF-beta. Although the protocol specified median TTP as an endpoint, median PFS was calculated given the US FDA guidance that PFS is preferred to TTP when the cause of death is likely due to cancer progression, as is the case in patients with GBM. PFS was calculated from the time of consent till either death or progression as per RANO criteria. Median PFS and OS were visualized through Kaplan–Meier survival curves^19^. Tumor responses and progression were evaluated using MRI per RANO criteria^20^. Efficacy evaluations were performed at eight weeks intervals or earlier if disease progression was suspected. Given the insufficient clinical responses observed which precluded assessment of association with circulating biomarkers, exploratory data on circulating VEGF levels were also investigated for mechanistic insights for design of further trials.

### Soluble VEGF levels

Double-spun, platelet-poor plasma-EDTA samples were collected from patients at baseline and on-treatment from all enrolling sites. After processing, samples were immediately frozen, shipped to Duke University Medical Center (Durham, NC, US), and stored at −80 °C until use. VEGF levels were measured using the CiraScan Multiplex platform (Aushon Biosystems Inc., Billerica, MA, US).

### Power calculation and statistical analysis

A one-stage accrual design with an accrual goal of 14 evaluable patients was initially planned to be employed to test the hypothesis that TRC105 can double the historical median time to progression in this population (i.e., from 1.5 months to 3.0 months). Assuming time to progression follows an exponential distribution, accrual takes approximately six months, and there is ≥6 months of additional follow-up once accrual has been completed, there would be 80% power to detect the specified difference using a two-sided test with 10% type I error. However, the protocol was amended. Therefore, a one-stage accrual design with an accrual goal of 22 evaluable patients was to be employed to test the hypothesis that TRC105 + bevacizumab could increase the median OS from 4 months to 7 months. Assuming OS followed an exponential distribution and an estimated accrual of approximately 12 months with 12 months of additional follow-up once accrual was completed, there would be 81% power to detect the specified difference using a two-sided test with 10% type I error. The safety population included all patients who received at least a portion of one dose of the study drug, TRC105. The evaluable population for determination of response included all patients with a baseline and a follow-up radiographic assessment for response at designated time points (e.g., every eight weeks). Descriptive statistics (mean, median, confidence intervals, and ranges for continuous data and percentages for categorical data) were used to summarize patient characteristics, treatment administration, safety, efficacy, and pharmacokinetic parameters.

### Reporting summary

Further information on research design is available in the Nature Portfolio Reporting Summary linked to this article.

## Results

A total of 22 patients with glioblastoma that had progressed after chemoradiation, and bevacizumab therapy were enrolled at four sites in the United States. 27% (6/22) were female. 9% (2/22) were African-American, 5% (1/22) were Asian and the rest (86%) were white (Table 1). The median age was 54.5 years (range 31–70). All 22 patients had measurable disease by modified RANO criteria at baseline. The median duration on the most recent VEGF-inhibitor-containing regimen prior to enrolling on this trial was 4.5 months.

**Table 1:** Baseline Patient Characteristics in the ENDOT trial.

Attributes	Values
Number of Patients, *N*	22
Age in years, Median (Range)	54.5 (31–70)
Gender, *N*(%)	
Female	6 (27%)
Male	16 (73%)
Tumor Type	
Glioblastoma	22 (100%)
Race	
Caucasian	19 (86.4%)
African-American	2 (9.1%)
Asian	1 (4.5%)_
Baseline Karnofsky Performance Status, N (%)	
60	4 (18%)
70	4 (18%)
80	10 (46%)
90	4 (18%)
Number of Prior Treatment Regimens, Median (Range)	3 (1–5)

As per the initial trial protocol, 6 patients were first enrolled in the monotherapy cohort of TRC105 10 mg/kg weekly, in 4-week cycles (cohort 1), with then primary outcome being time to progression (TTP). The median number of TRC105 doses received was 3.5. Overall survival was not captured in the monotherapy portion of the study. Five of six patients treated with TRC105 monotherapy had disease progression as per RANO criteria within two months of initiating treatment, and one patient treated with TRC105 alone discontinued based on the investigator’s decision related to overall clinical decline. Although the protocol initially specified TTP as the endpoint, PFS was calculated given the FDA guidance stipulating that PFS is preferred to TTP, when the cause of death is likely due to cancer progression, as is the case in GBM. The median PFS for the five evaluable monotherapy patients was 1.4 months, which was the same figure for median TTP in this cohort.

Due to a lack of activity in the patients treated with TRC105 monotherapy, the study was amended to an exploratory, non-randomized, open-label phase II clinical trial of TRC105, and a separate cohort added of bevacizumab plus TRC105, with the primary endpoint changed from median TTP to median overall survival (OS). 16 patients were enrolled in bevacizumab plus TRC105 in the combination portion of the study (cohort 2).

**Fig. 1:**
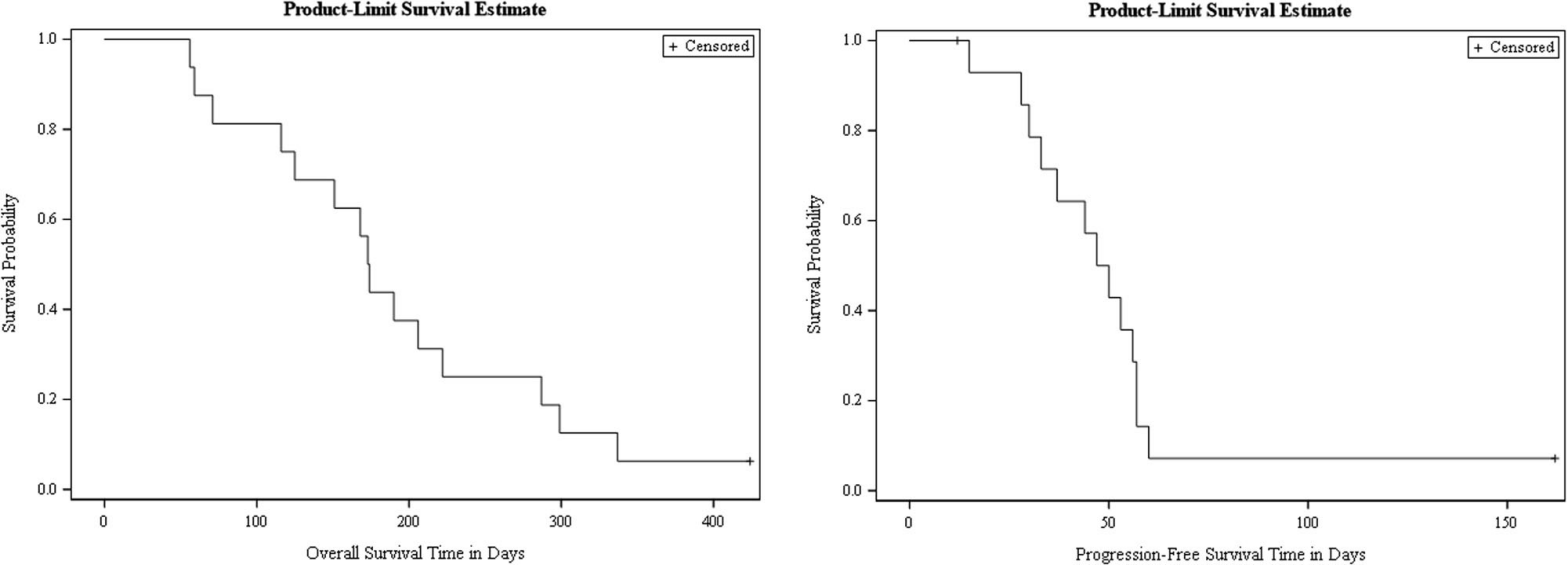
Kaplan–Meier curves of (left) overall survival and (right) progression-free survival in days for the TRC105 plus bevacizumab combination cohort (study cohort 2) of patients with GBM recurring after chemoradiation and bevacizumab.

In the TRC105 plus bevacizumab cohort, the median OS was calculated for 15 patients and found to be 5.7 months (95%CI 4.2–9.8) (Fig. 1A), since one patient in this cohort did not receive study intervention and had to be excluded from the efficacy analysis. Median OS in this cohort exceeded the OS of 4 months expected in GBM patients who progress following bevacizumab, based on historical data. However, enrollment in the combination portion of the study was terminated prior to full enrollment due to the study failing to meet pre-specified OS criteria of 7 months. Median PFS could be calculated based on 14 evaluable patients dosed bevacizumab+TRC105 and was found to be 1.8 months (95%CI 1.2–2.1) (Fig. 1B). PFS at 6 months was calculated based on 15 evaluable patients and was 13.3% (2/15). One patient treated with the combination had, just prior to trial enrollment, radiographic progression after four months of treatment with bevacizumab treatment. This patient was progression-free for six months on trial therapy, which was discontinued in cycle 7 based on investigator’s assessment. All other patients discontinued the study before completing six months of therapy. This patient was the only one with censored data other than 15 of the 16 patients enrolled in the combination cohort, who expired on study, or their expiry information was captured in follow-up. None of the patients had a reduction in tumor burden compared to baseline.

### Safety and tolerability

Overall, TRC105 was well tolerated both as a single agent and in combination with bevacizumab in patients with recurrent GBM. Most adverse events associated with TRC105 and/or bevacizumab were CTCAE grade 1 or 2 (Table 2). Headache (grade 1–3), epistaxis (grade 1–3), and fatigue (grade 1–3) were the most common study-medication-related adverse events (AEs) in both cohorts (Table 2). For cohort 2, headache occurred in 10 (46%) patients, epistaxis in 12 (55%) patients, and fatigue in 12 (55%) patients. Medication-related grade 3 AEs included epistaxis, headache, fatigue, TIA, lower leg edema possibly secondary to proteinuria, pulmonary embolism (PE), and sinusitis with periorbital cellulitis. Both PE and sinusitis occurred in the same patient. No grade 4 or 5 AEs were observed.

**Table 2:** Common (*N* > 1) Grade 1-2 and all serious (Grade 3–5) adverse events suspected to be associated with study medications, categorized by grade as per CTCAE v4.0 in both cohorts separately.

Toxicity (Preferred term)	Grade of toxicity					Attributes	
	Grade 1	Grade 2	Grade 3	Grade 4	Grade 5	*N*	Percentage^*^
Patients Administered Single Agent TRC105 (Cohort 1), Total *N* = 6							
Fatigue	1	2	0	0	0	3	(50%)
Headache	1	2	0	0	0	3	(50%)
Patients Administered TRC105 plus Bevacizumab (Cohort 2), Total *N* = 16							
Epistaxis	9	0	1	0	0	10	(63%)
Fatigue	4	4	1	0	0	9	(56%)
Headache	6	0	1	0	0	7	(44%)
Decreased appetite	2	1	0	0	0	3	(19%)
Flushing	3	0	0	0	0	3	(19%)
Gingival bleeding	3	0	0	0	0	3	(19%)
Rash	3	0	0	0	0	3	(19%)
Anemia	1	1	0	0	0	2	(13%)
Transient Ischemic Attack	0	1	1	0	0	2	(13%)
Dermatitis acneiform	2	0	0	0	0	2	(13%)
Infusion reaction	0	2	0	0	0	2	(13%)
Weight decreased	2	0	0	0	0	2	(13%)
Lower Leg Edema	0	0	1	0	0	1	(6.3%)
Pulmonary Embolism	0	0	1	0	0	1	(6.3%)
Periorbital cellulitis	0	0	1	0	0	1	(6.3%)
Sinusitis	0	0	1	0	0	1	(6.3%)

^*^All percentages rounded up to two significant digits.

Serious AEs that were considered unrelated to study drug treatment included grade 3 seizure, grade 3 increased cognitive impairment, grade 3 altered mental status, grade 2 seizure, grade 2 expressive aphasia, grade 3 hypoxia, and grade 3 cough. Unrelated AEs were primarily related to the central nervous system and considered by the investigators to reflect the primary diagnosis of GBM.

### Pharmacokinetics and immunogenicity

TRC105 was measurable above the target concentration of 25 ug/mL in all five patients who received single-agent TRC105 for whom data were available by cycle 1 day 15 (or cycle 1 day 22 in one case where sample acquisition was delayed by a week; mean trough concentration of 74 ug/mL; range 53–92 ug/mL). In patients who also received bevacizumab, TRC105 was measurable above the target concentration of 25 ug/mL in all 13 patients for whom data were available by cycle 1 day 15 (mean trough concentration of 96 ug/mL; range 58–179 ug/mL). Antibodies against TRC105 were detected in one of 12 patients for whom paired serum samples were available before dosing and at the end of the study.

### Serum VEGF levels

VEGF-A profiled was performed and data were available from multiple time points in two patients receiving TRC105 single-agent therapy. VEGF-A levels increased in both patients from 68 pg/mL to 93 pg/mL and from 62 pg/mL to 145 pg/mL, respectively, between baseline and cycle 1 day 15.

## Discussion

Recurrent glioblastoma (GBM) remains a highly challenging patient population. This multicenter, open-label exploratory trial of anti-endoglin inhibitor TRC105 (carotuximab), with and without VEGF inhibitor bevacizumab (ENDOT), demonstrated expected safety and tolerability in patients with glioblastoma that have progressed after chemoradiation and VEGF inhibitor therapy. Single-agent TRC105 was not found active for recurrent GBM, possibly because of increased plasma VEGF-A levels, which have been observed in other trials of TRC105 dosed as a single agent^21^. While the overall survival of 5.7 months in patients treated with TRC105 and bevacizumab may be interpreted as exceeding the OS of 4 months seen in GBM patients who progress following bevacizumab, based on historical data (6, 9–12); however, the observed median OS did not meet the pre-specified efficacy target of 7 months. Additionally, this novel therapy might have been too late in the recurrent setting, given the heavily pretreated cohort. The latter may have also represented a selection for patients with favorable outcomes, compared to the general glioblastoma patient population.

Endoglin (CD105) inhibition has been demonstrated to be of utility in advanced non-CNS tumors in several clinical trials. Rosen et al. reported outcomes from a clinical trial of TRC105 in 50 patients with advanced treatment-refractory solid tumors, including ten colorectal, nine prostate, five renal, and four lung cancer patients^22^. They found a maximum tolerated dose of 10 mg/kg weekly and 15 mg/kg biweekly. Nearly half of the enrolled patients were found to have stable disease or better response, indicating TRC105’s safety and feasibility in diverse tumors, including in the metastatic setting. Later, Karzai et al. reported outcomes, including angiogenic activity, of TRC105 in patients with metastatic castration-resistant prostate cancer. They reported ten patients with stable disease and eight having a decline in levels of prostate-specific antigen^21^. More recently, Choueiri and colleagues reported outcomes of TRC105 in combination with small-molecule tyrosine kinase inhibitor axitinib in metastatic renal cell carcinoma, a highly therapy-resistant setting. They found a partial response in nearly a third of patients with a median PFS of 11.3 months^23^.

This trial and its findings had several limitations, including its small sample size and the non-randomized design. A bevacizumab-only control arm would have been potentially useful comparator, its use by trial investigators was limited by funding constraints and because patients in the current trial had already failed bevacizumab. The trial was stopped early by the sponsor due to lack of demonstrated efficacy. However, this trial of an endoglin inhibitor combined with a VEGF inhibitor in GBM was remarkable for the fact that both vascularly-active drugs could be administered at their recommended Phase II doses (10 mg/kg each) without the development of tumor hemorrhage.

Consistent with prior studies of TRC105 and bevacizumab, the two drugs were well tolerated when given together in GBM patients. The expected adverse event profile of each drug was not potentiated when the two drugs were given together. Known toxicities of bevacizumab were observed at expected frequencies, with the exception that hypertension and proteinuria were rarely observed when bevacizumab was given with TRC105. Expected TRC105 toxicities of anemia, mucocutaneous telangiectasia (with resulting epistaxis and gingival bleeding), and low-grade infusion reactions were no more frequent than observed in studies of TRC105 given as a single agent^22^. One patient developed grade 3 epistaxis that resulted in a suspected serious adverse event of anemia, requiring hospitalization for transfusion. However, most cases of epistaxis observed were of grade 1, which is typical of TRC105 or bevacizumab treatment in other cancer types. Low-grade headache, a known adverse event of both TRC105 and bevacizumab treatment, was seen frequently, although attribution to the study drugs was confounded by the presence of underlying GBM.

In this trial, single-agent TRC105 resulted in increased serum levels of VEGF-A in recurrent glioblastoma, which may have potentially contributed to failure of single-agent therapy. This molecular finding gains value given prior mechanistic knowledge of upregulation of endoglin expression on tumor endothelial cells in response to VEGF inhibition (15,16). Given the lack of response, treatment of patients with TRC105 and bevacizumab before the development of resistance to bevacizumab appears to be a more promising approach to treating GBM patients. However, this approach was studied in the CTEP-sponsored trial NCT01648348 that compared bevacizumab to TRC105 with bevacizumab in bevacizumab-naïve recurrent GBM patients, and no improvement in PFS or OS was demonstrated^24^.

Future trials of TRC105 for glioblastoma, along with other novel investigational agents, should consider incorporating prospective serum VEGF-A profiling of patients. Individual patient-data (IPD) meta-analyses are warranted to increase analytical power in determining efficacy of investigational agents in neuro-oncology. Given the substantial challenges in drug discovery in neuro-oncology, incorporation of biomarkers as indicated by the present study is likely to be of considerable utility for the success of future trials^25^.

### Supplementary information

Supplementary Information

Reporting Summary

## Data Availability

The final amended study protocol is available in the supplementary material. This study, a clinical trial, generated individual patient data (IPD), which are protected and highly regulated in the US under Health Insurance Portability and Accountability Act (HIPAA). These IPD were used to generate Fig. 1 (Kaplan–Meier survival curves) along with Tables 1 and 2. Deidentified data is not being made available publicly given the low number of patients in the study (*N* = 22), the relatively low incidence of glioblastoma (compared to other systemic malignancies) and the novelty of the study medication, all of which together hinder assurance of patient confidentiality. IPD data, including source data for Fig. 1, informed consent form, and clinical study report, will be made available to qualified researchers upon reasonable request for IPD meta-analysis beginning at publication and ending 36 months following publication. Proposals should be directed to the corresponding author at manmeetA@baptisthealth.net.
