# Supplementary material for: Endoglin inhibitor TRC105 with or without bevacizumab for bevacizumab-refractory glioblastoma (ENDOT): a multicenter phase II trial
Source: Commun Med (Lond). 2023 Sep 8;3:120. doi: 10.1038/s43856-023-00347-0 (PMC10491825; doi:10.1038/s43856-023-00347-0)
Supplement: Supplementary file 1 — Supplementary Information [file 43856_2023_347_MOESM1_ESM.pdf]

CLINICAL PROTOCOL

Title:	A PHASE 2 EVALUATION OF TRC105 IN COMBINATION WITH BEVACIZUMAB FOR THE TREATMENT OF RECURRENT OR PROGRESSIVE GLIOBLASTOMA THAT HAS PROGRESSED ON BEVACIZUMAB
Protocol Number:	105GM201/Case 1312
Study Sponsor:	Cleveland Clinic<br>9500 Euclid Avenue<br>Cleveland, OH 44195<br>Phone: (216) 636-0007
Secondary Study Sponsor:	TRACON Pharmaceuticals, Inc.<br>8910 University Center Lane, Suite 700<br>San Diego, CA 92122<br>Phone: (858) 550-0780<br>Facsimile: (858) 550-0786
Medical Monitor:	Charles Theuer, MD PhD<br>8910 University Center Lane, Suite 700<br>San Diego, CA 92122<br>Office Phone: (858) 550-0780 x233<br>Fax: (858) 550-0786<br>Cell Phone: (858) 344-9400<br>Email: ctheuer@traconpharma.com
Signature & Date:	Signed: ______________________________ Dated: ________________

CLINICAL PROTOCOL	
Version Date:	Original version: January 26, 2012<br>Amendment #1: May 22, 2012<br>Amendment #2: December 17, 2012<br>Amendment #3: May 6, 2013<br>Amendment #4: August 15, 2013

1. SYNOPSIS

**Name of Sponsor/Company:** TRACON Pharmaceuticals, Inc.	
**Name of Investigational Product:** TRC105	
**Name of Active Ingredient:** TRC105	
**Title of Study:**<br>A PHASE 2 EVALUATION OF TRC105 IN COMBINATION WITH BEVACIZUMAB FOR THE TREATMENT OF RECURRENT OR PROGRESSIVE GLIOBLASTOMA THAT HAS PROGRESSED ON BEVACIZUMAB	
**Study Centers:** Cleveland Clinic (The study will be conducted at the main campus), Case Western University, Ohio State University and University of Cincinnati.	
**Principal Investigator:** Manmeet S. Ahluwalia, MD	
**Studied period (years):**<br>Date first patient enrolled: May 2012<br>Estimated date endpoint obtained: June 2015<br>Estimated date last patient completed: June 2015	**Phase of development:** 2
**Rationale:**<br>Angiogenesis plays a central role in the progression of solid cancer. TRC105 is an antibody to CD105, an important non-VEGF angiogenic target on proliferating endothelial cells. TRC105 inhibits angiogenesis, tumor growth and metastases in preclinical models. TRC105 has been well tolerated in patients with glioblastoma (GBM) as a single agent. The combination of	

TRC105 in combination with bevacizumab has demonstrated activity in bevacizumab-refractory cancer patients. We hypothesize that TRC105 when administered with bevacizumab will have activity in GBM patients who progress on bevacizumab. By targeting a non-VEGF pathway, TRC105 has the potential to complement VEGF inhibition by bevacizumab, which could represent a major advance in GBM therapy.

Objectives:

Primary:

- Determine median overall survival (OS) in patients with recurrent or progressive GBM who have progressed on bevacizumab.

Secondary:

- Assess safety and tolerability of TRC105 when given with bevacizumab by CTCAE version 4.0.
- Determine objective response rate (ORR) by modified RANO criteria.
- Determine the rate of progression free survival at 6 months (PFS-6).
- Determine the median time to progression.
- Explore associations between clinical outcome and soluble angiogenic biomarkers including but not limited to VEGF, PDGF and TGF-β

Methodology:

This is an open label single arm phase 2 trial. TRC105 will be administered at a dose of 10 mg/kg weekly on days 1, 8, 15 and 22 (except cycle 1 when dosing starts on day 8) and bevacizumab will be administered at a dose of 10 mg/kg on days 1 and 15 of each 28-day cycle. TRC105 dosing will begin on cycle 1 day 8 and will be split into two doses with 3 mg/kg administered on cycle 1 day 8 and the balance of the weekly dose administered on cycle 1 day 11. The entire weekly dose of TRC105 will then be given on cycle 1 day 15 and weekly thereafter.

Number of Patients:

A one stage accrual design with an accrual goal of 22 evaluable patients will be employed in order to test the hypothesis that TRC105 + bevacizumab can increase the overall survival from 4 months to 7 months. Assuming an approximately 10 % exclusion rate an additional 4 patients may be entered to replace ineligible patients or patients who withdraw consent prior to receiving study treatment. Assuming overall survival follows an exponential distribution, accrual takes approximately 12 months, and that there is 12 months of additional follow-up once accrual has been completed, there will be 81% power to detect the specified difference using a two-sided test with 10 % type I error. Approximately 125-150 patients with recurrent GBM are seen each year at Cleveland Clinic and Case Western University. It is estimated that 2-3 patients will be enrolled per month and that the study will complete enrollment over a 10-12 month time frame.

Eligibility Criteria:

Inclusion criteria:

1. Patients with histologically confirmed glioblastoma, recurrent after prior external-beam fractionated radiotherapy and temozolomide chemotherapy.
2. Patients with documented radiographic progression following bevacizumab therapy for treatment of glioblastoma.
3. Patients with up to 3 prior recurrences are allowed.
4. Karnofsky performance status ≥ 70%.
5. Age ≥ 18 years old.
6. Patients must have the following laboratory values:
   - Absolute neutrophil count (ANC) ≥ 1.5 x 10^9/L
   - Platelets ≥ 100 x 10^9/L
   - Hemoglobin (Hgb) > 9 g/dL
   - Serum total bilirubin: ≤ 1.5 x ULN
   - ALT and AST ≤ 3.0 x ULN
   - Serum creatinine ≤ 1.5 x ULN
   - Blood coagulation parameters: INR ≤ 1.5
7. Minimum interval since completion of radiation treatment is 12 weeks

8. Minimum interval since last drug therapy:
    - 2 weeks since last non-cytotoxic therapy
    - 3 weeks must have elapsed since the completion of a non-nitrosourea-containing chemotherapy regimen
    - 6 weeks since the completion of a nitrosourea-containing chemotherapy regimen.
9. Patients must have signed an approved informed consent and authorization permitting release of personal health information.
10. Patients with the potential for pregnancy or impregnating their partner must agree to follow acceptable birth control methods to avoid conception. The anti-proliferative activity of this experimental drug may be harmful to the developing fetus or nursing infant. Female patients of child-bearing potential must have a negative pregnancy test.
11. Patients must have no concurrent malignancy except curatively treated basal or squamous cell carcinoma of the skin or carcinoma in situ of the cervix and breast, adequately treated stage I or II cancer from which the patient is in complete remission. Patients with other prior malignancies must be disease-free for ≥ three years.
12. Patients must be maintained on a stable or decreasing corticosteroid regimen from the time of their baseline scan until the start of treatment and/or for at least 5 days before starting treatment.
13. Patients must have a Mini Mental State Exam score ≥ 15.

<u>Exclusion criteria:</u>

1. Patients who have had previous treatment with TRC105.
2. Patients who have undergone major surgery (e.g. intra-thoracic, intra-abdominal or intra-pelvic), open biopsy or significant traumatic injury ≤ 4 weeks prior to starting study drug, or patients who have had minor procedures, percutaneous biopsies or placement of vascular access device ≤ 1 week prior to starting study drug, or who have not recovered from side effects of such procedure or injury
3. Patients with impaired cardiac function or clinically significant cardiac diseases, including any of the following:
    - History or presence of serious uncontrolled ventricular arrhythmias
    - Clinically significant resting bradycardia
    - Any of the following within 6 months prior to starting study drug: myocardial infarction (MI), severe/unstable angina, Coronary Artery Bypass Graft (CABG),

Congestive Heart Failure (CHF), Cerebrovascular Accident (CVA), Transient Ischemic Attack (TIA), Pulmonary Embolism (PE)
    - Uncontrolled hypertension (defined by a SBP ≥ 160 mm Hg or DBP ≥ 100 mm Hg while on anti-hypertensive medications)

4. Patients with cirrhosis, or active viral or nonviral hepatitis.
5. Known diagnosis of human immunodeficiency virus (HIV) infection (HIV testing is not mandatory)
6. Other concurrent severe and/or uncontrolled concomitant medical conditions (e.g. active or uncontrolled infection, uncontrolled diabetes) that could cause unacceptable safety risks or compromise compliance with the protocol
7. Pregnant or breast-feeding women
8. Patients with known hypersensitivity to Chinese hamster ovary cell products or other recombinant human, chimeric, or humanized antibodies.
9. Patients with active bleeding or pathologic conditions that carry a high risk of bleeding, (i.e. hereditary hemorrhagic telangiectasia).
10. Patients who are currently receiving anticoagulation treatment
11. Patients unwilling or unable to comply with the protocol

TRC105 Dose and Route of Administration:

Following the appropriate premedication regimen, TRC105 dosing will begin on cycle 1 day 8 and will be split into two doses: 3 mg/kg of TRC105 will be administered on cycle 1 day 8 over a period of 4 hours and 7 mg/kg will be administered on cycle 1 day 11 infused over a period of 4 hours. The full 10 mg/kg TRC105 dose will be administered on cycle 1 day 15 infused over 4 hours. For patients who complete at least one 4 hour infusion without the development of any infusion reactions, the subsequent TRC105 infusion may be reduced to 2 hours. For patients who complete a 2 hour infusion without the development of any infusion reactions, subsequent TRC105 infusions may be reduced to 1 hour.

Duration of Treatment:

Patients are eligible for treatment with bevacizumab and TRC105 until disease progression or unacceptable toxicity. A patient should be withdrawn from study treatment if, in the opinion of the Investigator, it is medically necessary, or if it is the wish of the patient. In addition, patients

will be withdrawn from treatment in the case of:

1. Modified RANO criteria defined disease progression. In cases where RANO cannot be applied, progression should be based on unequivocal evidence of progressive disease sufficient to require a change in therapy.
2. A need for surgery, radiation, or for other anticancer therapy not specified in the protocol.
3. TRC105 dose delay > 8 weeks.
4. Lost to follow-up or noncompliant.
5. Pregnancy. Pregnant patients should be followed for the duration of the pregnancy and the outcome of the pregnancy should be documented.
6. Arterial thrombosis of any grade (including cerebrovascular ischemia, cardiac ischemia/infarction, or peripheral or visceral arterial ischemia) or grade 3 or 4 venous thrombosis (including pulmonary embolism).

Parameters to be Assessed:

Safety:

Safety assessments will include physical exams, performance status, laboratory results (complete blood counts and serum chemistry) and 12-lead ECG's (in patients who develop arrhythmia), and additional studies as clinically indicated. A formally chartered in-house TRACON Safety Review Team will review safety data. In addition, recurring teleconferences will be held with Investigators at all clinical sites.

Pharmacokinetics:

Serum TRC105 concentrations will be measured using validated methods at time points specified in the schedule of events.

Immunogenicity:

Immunogenicity (HAMA/HACA) will be measured using validated methods at time points specified in the schedule of events.

Exploratory Biomarkers:

Serum concentrations of a panel of angiogenic protein biomarkers will be measured at baseline,

during treatment, and at the time of progression to explore TRC105 pharmacodynamics.

Efficacy:

Modified RANO criteria will be applied to assess response and progression.

Funding, Regulatory, and Feasibility Issues:

TRC105 study drug will be provided by TRACON. Commercial bevacizumab will be utilized in this study. Up to 26 patients will be enrolled for a total of 22 evaluable patients. Every year 125-150 patients with recurrent GBM are seen at Cleveland Clinic and at Case Western Reserve University. Approximately 2-3 patients per month will be entered onto the study and accrual will be completed in 10-12 months. All patients will be followed until death.

Patient Acceptability/Ethics and Consent Issues:

There are no effective therapies for patients with recurrent GBM that progresses on bevacizumab therapy. Bevacizumab is frequently continued in these patients for lack of better options. TRC105 has shown evidence of clinical activity in patients with bevacizumab-refractory cancers when combined with bevacizumab, and therefore this combination treatment deserves study in patients with refractory GBM.

TABLE OF CONTENTS, LIST OF TABLES, AND LIST OF FIGURES

TABLE OF CONTENTS

1. SYNOPSIS 3
TABLE OF CONTENTS, LIST OF TABLES, AND LIST OF FIGURES 10
2. INTRODUCTION 22
2.1. Background....................22
2.1.1. Glioblastoma Multiforme 22
2.1.2. Angiogenesis and Cancer 22
2.1.2.1. Angiogenesis and Glioblastoma 23
2.1.3. CD105 and Angiogenesis 23
2.1.4. TRC105 Background....................25
2.1.4.1. Phase 1 Monotherapy Study Design for Solid Cancers**Error! Bookmark not defined.**
2.1.4.2. Summary from Phase 1b 105ST102 TRC105 + Bevacizumab: Cohorts 1- 5**Error! Bookmark not defined.**
2.1.4.3. Additional Studies with TRC105....................**Error! Bookmark not defined.**
2.1.5. Summary from Phase 2 trial of TRC105 Monotherapy in Recurrent or Progressive Glioblastoma (original protocol): ....**Error! Bookmark not defined.**
2.1.6. Population to be Studied and Rationale....................**Error! Bookmark not defined.**
2.1.7. Potential Risks and Benefits to Human Patients....................25
2.1.7.1. Potential Risks 25
2.1.7.2. Potential Benefits....................25
2.1.8. Justification of the Dose, Schedule and Route of Administration of TRC105....................25
2.1.9. Conduct....................25
3. TRIAL OBJECTIVES AND PURPOSE....................26

3.1. Primary objectives 26
3.2. Secondary objectives 26
4. INVESTIGATIONAL PLAN 27
4.1. Overall Study Design and Plan 27
4.1.1. Overview .... **Error! Bookmark not defined.**
4.1.2. Trial Procedures .... **Error! Bookmark not defined.**
4.1.2.1. Screening .... **Error! Bookmark not defined.**
4.1.2.2. Trial Period .... **Error! Bookmark not defined.**
4.1.3. End of Study Assessments .... **Error! Bookmark not defined.**
4.1.4. Post Treatment Follow-up .... **Error! Bookmark not defined.**
5. SELECTION AND WITHDRAWAL OF PATIENTS 28
5.1. Patient Inclusion Criteria 28
5.2. Exclusion Criteria: 29
5.3. Patient Withdrawal Criteria 30
6. TREATMENT OF PATIENTS 31
6.1. Description of TRC105 Study Drug 31
6.2. Composition of TRC105 31
6.3. TRC105 Dose Level 31
6.4. TRC105 Packaging and Labeling 31
6.5. TRC105 Storage and Shipping 31
6.6. TRC105 Preparation 31
6.7. TRC105 Administration 31
6.7.1. TRC105 Dose Reduction .... **Error! Bookmark not defined.**
6.7.2. Management of TRC105 Infusion Reactions .... **Error! Bookmark not defined.**

6.8. TRC105 Study Drug Accountability 31
6.9. TRC105 Study Drug Handling and Disposal 31
6.10. Bevacizumab Packaging 31
6.11. Bevacizumab Preparation 31
6.12. Bevacizumab Administration 31
6.12.1. Bevacizumab Dose Modification 32
6.13. Bevacizumab Drug Accountability 32
6.14. Bevacizumab Handling and Disposal 32
6.15. Concomitant Medications 32
6.16. TRC105 Study Drug Treatment Compliance 32
6.17. Patient Enrollment 32
7. ASSESSMENT OF EFFICACY 33
7.1. Response Assessment 33
8. ASSESSMENT OF SAFETY 37
8.1. Safety Parameters 37
8.1.1. Laboratory Safety Assessments 37
8.1.1.1. Hematology, Serum Chemistry, Coagulation, Pregnancy Test 37
8.1.1.2. Urinalysis 37
8.1.1.3. Physical Examination 38
8.1.1.4. Vital Signs 38
8.1.1.5. Performance Status 38
8.1.1.6. ECG 38
8.2. Adverse Events 38
8.2.1. Definition of Adverse Event 38

8.2.2. Serious Adverse Events 39
8.2.2.1. Hospitalization 40
8.3. Reporting Adverse Events 41
8.3.1. Eliciting Adverse Event Information 41
8.3.2. Adverse Event Reporting Period 41
8.3.3. Reporting Requirements 41
8.3.4. Recording Adverse Events in the Case Report Forms 42
8.3.5. Grading of Adverse Event Severity 42
8.3.6. Relationship to TRC105 Study Drug 42
8.3.7. Expectedness 43
8.3.8. Exposure *in Utero* 43
8.3.9. Follow-up of Unresolved Adverse Events 44
8.4. Safety Monitoring 44
9. OTHER LABORATORY ASSESSMENTS 45
9.1. Pharmacokinetics 45
9.1.1. Predose TRC105 Concentration 45
9.1.2. End of Infusion TRC105 Concentrations 45
9.2. TRC105 Immunogenicity 45
9.3. Protein Biomarkers 45
10. STATISTICS 46
10.1. Definition of Analyzed Study Populations 46
10.2. Data Analysis 46
10.2.1. Analysis of Efficacy and Safety 47
10.2.2. Analysis of Protein Biomarkers 47

10.2.3. Analysis of Pharmacokinetics....47
10.2.4. Analysis of Immunogenicity....47
11. DIRECT ACCESS TO SOURCE DATA/DOCUMENTS....49
12. QUALITY CONTROL AND QUALITY ASSURANCE....50
13. ETHICS....51
13.1. Institutional Review Board (IRB)/Independent Ethics Committee (IEC)....51
13.2. Ethical Conduct of the Study....51
13.3. Written Informed Consent....51
13.4. Patient Compensation....51
14. DATA HANDLING AND RECORDKEEPING....52
14.1. Inspection of Records....52
14.2. Retention of Records....52
15. DEFINITION OF END OF TRIAL....54
15.1. End of Trial in all Participating Countries....54
15.2. TRACON Discontinuation Criteria....54
16. PUBLICATION OF TRIAL RESULTS....55
17. FINANCING AND INSURANCE....56
18. INVESTIGATOR PROTOCOL AGREEMENT: 105GM201....57
19. REFERENCES....58
20. APPENDICES....63
20.1. Appendix 1: National Cancer Institute (NCI) Common Terminology Criteria for Adverse Events (CTCAE) 63
20.2. Appendix 2: Karnofsky performance scale....64
20.3. Appendix 3: New York Heart Association Functional Capacity Classification....65
20.4. Appendix 4: Bevacizumab Package Insert....66

LIST OF TABLES

Table 1: Abbreviations and Specialist Terms 17
Table 2: 105ST101 Phase 1 Summary of Infusion Reactions (N=50)**Error! Bookmark not defined.**
Table 3: 105ST101 Phase 1 Possibly Related Adverse Events (N=50)**Error! Bookmark not defined.**
Table 4: Schedule of Assessments ....**Error! Bookmark not defined.**
Table 5: Recommended Dexamethasone Taper Schedule ...Error! Bookmark not defined.
Table 6: Allowable TRC105 Dose Modifications ....**Error! Bookmark not defined.**
Table 7: Management of TRC105 Infusion Reactions ....**Error! Bookmark not defined.**
Table 8: RANO Definitions of Tumor Response 34
Table 9: Modified RANO Response Evaluation Criteria 36
Table 10: Adverse Event Grading....................42

LIST OF FIGURES

Figure 1: Inhibition of VEGF-Induced HUVEC Sprouting by TRC105 and Bevacizumab**Error! Bookmark not defined.**
Figure 2: TRC105 Pharmacokinetic Data from Study 105ST101 .**Error! Bookmark not defined.**

Table 1: Abbreviations and Specialist Terms

Abbreviation or specialist term	Explanation
ADCC	Antibody-Dependent Cell-mediated Cytotoxicity
AE	Adverse Event
AFP	Alpha Fetoprotein
AIDS	Acquired Immunodeficiency Syndrome
ALKs	Activin receptor-Like Kinases
ALT	Alanine Aminotransferase
ANC	Absolute Neutrophil Count
AST	Aspartate Aminotransferase
AUC	Area Under the Curve
AUC_{last}	Time of Last Measurable Concentration of Area Under the Curve
BALB/c mice	Mouse Strain
BUN	Blood Urea Nitrogen
CA-125	Cancer Antigen-125
CABG	Coronary Artery Bypass Graft
CBC	Complete Blood Count
CEA	Carcinoembryonic Antigen
CHOP	Cyclophosphamide Hydroxydaunomycin Oncovin® Prednisone
CL	Clearance
C_{max}	Maximum Serum Concentration
CPA	Cyclophosphamide

Abbreviation or specialist term	Explanation
CR	Complete Response
CRF	Case Report Form
CT	Computed Tomography
CTC	Common Terminology Criteria
dL	Deciliter
DLT	Dose Limiting Toxicity
DVT	Deep Vein Thrombosis
ECG	Electrocardiogram
ECM	Extracellular Matrix
EGFR	Epidermal Growth Factor Receptor
ELISA	Enzyme-Linked ImmunoSorbent Assay
EOS	End of Study
FDA	Food and Drug Administration
g	Gram
GCP	Good Clinical Practice
HACA	Human Anti-Chimeric Antibodies
HAMA	Human Anti-Murine Antibodies
Her-2	Human epidermal growth factor receptor 2
HHT-1	Hereditary Hemorrhagic Telangiectasia Type 1
HIF-1-α	Hypoxia-Inducible Factor-1-α
HIPAA	Health Insurance Portability and Accountability Act

Abbreviation or specialist term	Explanation
HIV	Human Immunodeficiency Virus
HRA	Health Regulatory Authority
HUVECs	Human Umbilical Vein Endothelial Cells
ICH	International Conference on Harmonization
ID	Identification
IEC	Independent Ethics Committee
IgG	Immunoglobulin G
IgM	Immunoglobulin M
INR	International Normalized Ratio
IP	Intraperitoneal
IRB	Institutional Review Board
i.v.	Intravenous
K_d	Avidity Binding Constant
kg	Kilogram
L	Liter
LDH	Lactate Dehydrogenase
LOQ	Limit of Quantification
µL	Microliter
Mg	Milligram
mL	Milliliter
MACA	Monkey Anti-Chimeric Antibody

Abbreviation or specialist term	Explanation
MAMA	Monkey Anti-Murine Antibody
MI	Myocardial Infarction
mm	Millimeter
MRI	Magnetic Resonance Imaging
MTD	Maximum Tolerated Dose
NCI	National Cancer Institute
NCIC	National Cancer Institute of Canada
ng	Nanogram
NHP	Nonhuman Primate
NOAEL	No Adverse Effect Level
PBS	Phosphate-Buffered Saline
PD	Progressive Disease
PDGF	Platelet Derived Growth Factor
PlGF	Placental Growth Factor
pM	Picomolar
PR	Partial Response
PSA	Prostate Specific Antigen
PT	Prothrombin Time
PTCA	Percutaneous Transluminal Coronary Angioplasty
PTT	Partial Thromboplastin Time
RANO	Response Assessment in Neuro-Oncology

Abbreviation or specialist term	Explanation
RECIST	Response Evaluation Criteria in Solid Tumors
SAE	Serious Adverse Event
sCD105	Soluble CD105/endoglin
SCID	Severe Combined Immunodeficient
SD	Stable Disease
SGOT	Serum Glutamic Oxaloacetic Transaminase
SGPT	Serum Glutamic Pyruvic Transaminase
SN6j	Murine parent antibody of TRC105
sVEGFR2	Soluble VEGF Receptor 2
TGF-β	Transforming Growth Factor
TSH	Thyroid Stimulating Hormone
ULN	Upper Limit of Normal
US	United States of America
VEGF	Vascular Endothelial Growth Factor

2. INTRODUCTION

2.1. Background

2.1.1. Glioblastoma Multiforme

Up until the early 2000's, the standard of care for glioblastoma (GBM) was surgical resection followed by radiation therapy. In 2005, Stupp et al. demonstrated that by adding temozolomide during and after radiation therapy, the median survival of patients with GBM improved from 12.1 months to 14.6 months [1]. As a result, temozolomide with radiation became the standard of care for the treatment of GBM. Despite this significant improvement, nearly all patients with GBM recur with a median time to progression of approximately 6 months [1]. More than 70% of participants survive less than 2 years, and fewer than 10% are alive at 5 years following diagnosis[1].

2.1.2. Angiogenesis and Cancer

Angiogenesis is required for the survival and growth of solid cancers [2, 3]. It is generally accepted that solid cancers have two phases, an avascular phase and a vascular phase [3]. During the initial avascular phase, tumors exist as small aggregates of malignant cells supported by simple diffusion of oxygen and nutrients. The progressive growth of solid cancers beyond clinically occult sizes requires the continuous formation of new blood vessels, a process known as tumor angiogenesis. Tumor growth and metastasis require angiogenesis. Therefore, inhibition of tumor angiogenesis and selective inhibition of the tumor vasculature represent potentially effective strategies for the prevention and treatment of solid cancers.

Therapies directed against targets implicated in the development of tumor angiogenesis are attractive for many reasons. First, except for female reproduction and wound healing, angiogenesis in adults is generally part of a pathologic process such as tumor growth or choroidal neovascularization. Second, treatments that interrupt tumor angiogenesis should apply broadly to all solid cancers. Third, angiogenic targets are present in the plasma or on endothelial cells themselves. These targets are readily accessible to antibody treatments, in contrast to targets expressed within tumors that are more difficult for antibodies to access. Fourth, angiogenic targets on vascular endothelial cells are less prone to genetic mutation than targets expressed by genetically unstable cancer cells. As a result, development of resistance may be more predictable for agents that target endothelial cell functions than for those targeting cancer cells.

Indeed, agents that target pathways required for tumor angiogenesis have an important role in the therapy of cancer patients. The monoclonal antibody bevacizumab, which binds to the angiogenic cytokine VEGF, significantly prolongs overall survival for patients with advanced colorectal cancer or non-small cell lung cancer when added to standard chemotherapy regimens [4, 5]. Bevacizumab is also effective therapy for renal cell cancer, breast cancer and malignant glioma [6-8]. Orally available small molecule VEGF inhibitors

include sunitinib, sorafenib, pazopanib and axitinib, all of which have been shown to prolong survival in patients with metastatic renal cell cancer and/or hepatocellular cancer [8-11].

2.1.2.1. Angiogenesis and Glioblastoma

Antiangiogenic strategies are of interest in treating GBM due to the highly vascular nature of these tumors. Preclinical data have demonstrated the dependence of glioma growth on generation of tumor-associated blood vessels [12, 13]. The malignant cells of GBM express high levels of vascular endothelial growth factor (VEGF) in situ, and inhibition of VEGF signaling delays the growth of glioma xenografts in immunodeficient mice [14]. Bevacizumab (Avastin; Genentech, South San Francisco, CA) is a humanized monoclonal antibody that targets VEGF and has clinical activity in a number of human tumors, including colorectal cancer and non–small-cell lung cancer [5,15]. Bevacizumab has limited single-agent activity in these cancer types and is generally given in combination with cytotoxic agents. The BRAIN study, a phase II randomized trial evaluated the role of bevacizumab (alone or in combination with irinotecan) in 167 patients with recurrent GBM [7]. The progression free survival (PFS) at 6 months (PFS-6) was 42.6% and 50.3%, objective response rate (ORR) was 28.2% and 37.8% and median OS was 9.2 months and 8.7 months in the monotherapy and combination arms, respectively. In a study done at the National Cancer Institute (NCI), 48 patients with recurrent GBM were treated with bevacizumab producing a response rate (RR) of 35%, PFS-6 of 29% and a median OS of 31 weeks [16]. Clinical benefit was evident as decreasing cerebral edema, tapering steroid doses and improvement in neurological function in nearly half of the patients. Addition of irinotecan to patients who progressed following bevacizumab did not provide any additional benefit. The FDA recently approved bevacizumab in patients with recurrent GBM based on single-agent response rates [17]. However, the duration of effect of bevacizumab appears to be limited and there are growing concerns about the long-term efficacy of bevacizumab. To date, clinical trials with bevacizumab with recurrent glioblastoma have shown an improvement of PFS without substantial improvement in OS. Furthermore, there is no effective therapy for patients with recurrent GBM after progression on bevacizumab. Various studies have reported PFS of 5-8 weeks and OS of 3-5 months emphasizing the dismal prognosis of these patients and an urgent need for options in this setting of bevacizumab-refractory recurrent GBM [16,18-21]; thus, there still remains a great need for more effective agents. We hypothesize that anti-angiogenic approaches directed against non-VEGF endothelial targets will inhibit angiogenesis, even after the GBM has developed resistance to therapies directed against VEGF and its receptors.

2.1.3. CD105 and Angiogenesis

CD105 (endoglin) is a homodimeric cell membrane glycoprotein that was initially identified as a human leukemia-associated antigen [22] and later also found on endothelial cells [23, 24]. The expression of CD105 is relatively restricted in adult humans, and high levels of membrane CD105 in adult humans is limited to proliferating endothelial cells and red blood cell precursors known as proerythroblasts [25]. CD105 is a TGF-β coreceptor that is essential for angiogenesis [26, 27] and CD105 is strongly expressed on the proliferating vascular endothelium of solid tumors [28, 29]. All of these properties make CD105 an attractive target for the antiangiogenic therapy of cancer [30].

Vascular targeted therapy may more effectively address large established tumors than conventional antiangiogenic therapies that target receptor ligands such as anti-VEGF therapy [31]. In animal models, CD105 targeted therapy has demonstrated both vascular targeting effects and antiangiogenic effects, as it induces regression of established tumors, prevents new tumor formation, and inhibits the expansion of existing tumors [29, 32-35]. Therefore, CD105 is a novel alternative target relative to currently available angiogenesis inhibitors, all of which target the VEGF pathway.

CD105 alters downstream signaling of multiple kinase receptor complexes of the TGF-β superfamily, including TGF-β receptors, activin receptor-like kinases (ALKs) and activin receptors [36]. In the absence of CD105, activation of TGF-β receptors results in phosphorylation of SMAD proteins that inhibit endothelial cell growth. In the presence of CD105, activation of TGR-β receptors results in phosphorylation of SMAD proteins that promote endothelial cell growth. Moreover, anti-CD105 antibody therapy acts synergistically with TGF-β to inhibit endothelial cell proliferation and angiogenesis [37].

CD105 expression is required for endothelial cell proliferation, and CD105 is upregulated in the setting of hypoxia through the induction of hypoxia-inducible factor-1-α (HIF-1-α) [38]. CD105 has also been shown to protect hypoxic cells from apoptosis [39]. Targeted inactivation (knockout) of murine CD105 results in defective vascular development. Mice lacking CD105 die *in utero* from defective vascular development by gestational day 11 [27].

CD105 is critical for normal human blood vessel development [40]. CD105 haplotype insufficiency causes a well-described syndrome known as hereditary hemorrhagic telangiectasia type 1 (HHT-1 or Rendu-Osler-Weber Syndrome). HHT-1 is a rare autosomal dominant genetic disorder characterized by localized angiodysplasia involving the nasal, buccal, gastrointestinal mucosa and skin microvasculature. Angiodysplasia also occurs in vessels from internal organs including the lungs, liver and brain [41]. The genotype is manifested *in utero*, but the phenotype does not become apparent for many years following birth. Affected patients commonly present with epistaxis in the second decade of life. The phenotype of this disorder is limited to vascular effects, indicating the specific role of CD105 in angiogenesis [42].

CD105 is highly expressed on the proliferating endothelial cells of tumor vessels including brain, lung, breast, colorectal, gastric, liver, endometrial, renal cell, head and neck, and ovarian cancers. In adults, CD105 expression is limited to vascular endothelial and stromal cells and proerythroblasts, a red blood cell precursor [25].

Importantly, CD105 expression is upregulated in tumor endothelial cells following inhibition of the VEGF pathway. CD105 expression increased more than 2-fold in human pancreatic cancers grown in mice treated with an antibody that binds VEGF [43]. As well, treatment of human bladder cancers grown in mice with an antibody that blocks activation of the VEGF receptor increased CD105 expression within the core tumor vasculature [44].

CD105 expression is a prognostic factor in solid tumor patients. In studies from the United States and Europe, high CD105 microvessel density was shown to be an independent marker for worse survival in patients with malignant glioma [45]. In addition, high microvessel density by CD105 has been correlated with poor prognosis in clinical studies of breast cancer [46, 47], ovarian cancer [48, 49], lung

cancer [50], prostate cancer [51, 52], colorectal cancer [53, 54], gastric cancer [55], endometrial cancer [56], hepatocellular carcinoma [56, 57], esophageal adenocarcinoma [58], and head and neck cancer [59, 60].

Plasma CD105 levels measured by sandwich ELISA are prognostic in retrospective studies of cancer patients. In one study, the mean plasma CD105 concentration in 76 patients with colorectal cancer was 4-fold higher than the mean value in 40 healthy subjects without cancer [53]. In the study, a positive correlation was observed between CD105 concentration and stage of disease. In another study, the mean sCD105 concentration in 59 patients with advanced metastatic solid cancer was 63.8 ng/mL versus 41.0 ng/mL in cancer patients without metastases, and 28.3 ng/mL in patients without a cancer diagnosis [61]. In a study of breast cancer patients receiving hormonal therapy, the upper limit of normal for soluble CD105 was determined to be 8.70 ng/mL, and patients with elevated CD105 had shorter overall survival than those who did not [47].

2.1.4. TRC105 Background

REDACTED

2.1.5. Potential Risks and Benefits to Human Patients

2.1.5.1. Potential Risks

REDACTED

2.1.5.2. Potential Benefits

REDACTED

2.1.6. Justification of the Dose, Schedule and Route of Administration of TRC105

REDACTED

2.1.7. Conduct

The clinical trial will be conducted in compliance with the protocol, and the applicable regulatory requirements.

3. TRIAL OBJECTIVES AND PURPOSE

3.1. Primary objectives

- Determine median overall survival (OS) in patients with recurrent or progressive GBM who have progressed on bevacizumab.

3.2. Secondary objectives

- Assess safety and tolerability of TRC105 when given with bevacizmab by CTCAE version 4.0.
- Determine objective response rate (ORR) by modified RANO criteria.
- Determine progression free survival at 6 months (PFS-6).
- Determine the median time to progression
- Explore associations between clinical outcome and soluble angiogenic biomarkers including but not limited to VEGF, PDGF and TGF-β.

4. INVESTIGATIONAL PLAN

4.1. Overall Study Design and Plan

REDACTED

5. SELECTION AND WITHDRAWAL OF PATIENTS

5.1. Patient Inclusion Criteria

1. Patients with histologically confirmed glioblastoma, recurrent after prior external-beam fractionated radiotherapy and temozolomide chemotherapy.
2. Patients with documented radiographic progression following bevacizumab therapy for treatment of glioblastoma .
3. Patients with up to 3 prior recurrences are allowed.
4. Karnofsky performance status ≥ 70%.
5. Age ≥ 18 years old.
6. Patients must have the following laboratory values:
   - Absolute neutrophil count (ANC) ≥ 1.5 x 10^9/L
   - Platelets ≥ 100 x 10^9/L
   - Hemoglobin (Hgb) > 9 g/dL
   - Serum total bilirubin: ≤ 1.5 x ULN
   - ALT and AST ≤ 3.0 x ULN
   - Serum creatinine ≤ 1.5 x ULN
   - Blood coagulation parameters: INR ≤ 1.5
7. Minimum interval since completion of radiation treatment is 12 weeks
8. Minimum interval since last drug therapy:
   - 2 weeks since last non-cytotoxic therapy
   - 3 weeks must have elapsed since the completion of a non-nitrosourea containing chemotherapy regimen
   - 6 weeks since the completion of a nitrosourea containing chemotherapy regimen.
9. Patients must have signed an approved informed consent and authorization permitting release of personal health information.
10. Patients with the potential for pregnancy or impregnating their partner must agree to follow acceptable birth control methods to avoid conception. The anti-proliferative activity of this experimental drug may be harmful to the developing fetus or nursing infant. Female patients of child-bearing potential must have a negative pregnancy test.
11. Patients must have no concurrent malignancy except curatively treated basal or squamous cell carcinoma of the skin or carcinoma in situ of the cervix and breast, adequately treated stage I or II cancer from which the patient is in complete remission. Patients with other prior malignancies must be disease-free for ≥ three years.

12. Patients must be maintained on a stable or decreasing corticosteroid regimen from the time of their baseline scan until the start of treatment and/or for at least 5 days before starting treatment.
13. Patients must have a Mini Mental State Exam score ≥ 15.

5.2. Exclusion Criteria:

1. Patients who have had previous treatment with TRC105.
2. Patients who have undergone major surgery (e.g. intra-thoracic, intra-abdominal or intra-pelvic), open biopsy or significant traumatic injury ≤ 4 weeks prior to starting study drug, or patients who have had minor procedures, percutaneous biopsies or placement of vascular access device ≤ 1 week prior to starting study drug, or who have not recovered from side effects of such procedure or injury
3. Patients with impaired cardiac function or clinically significant cardiac diseases, including any of the following:
    - History or presence of serious uncontrolled ventricular arrhythmias
    - Clinically significant resting bradycardia
    - Any of the following within 6 months prior to starting study drug: myocardial infarction (MI), severe/unstable angina, Coronary Artery Bypass Graft (CABG), Congestive Heart Failure (CHF), Cerebrovascular Accident (CVA), Transient Ischemic Attack (TIA), Pulmonary Embolism (PE)
    - Uncontrolled hypertension (defined by a SBP ≥ 160 mm Hg or DBP ≥ 100 mm Hg with anti-hypertensive medications)
4. Patients with cirrhosis, or active viral or nonviral hepatitis.
5. Known diagnosis of human immunodeficiency virus (HIV) infection (HIV testing is not mandatory)
6. Other concurrent severe and/or uncontrolled concomitant medical conditions (e.g. active or uncontrolled infection, uncontrolled diabetes) that could cause unacceptable safety risks or compromise compliance with the protocol
7. Pregnant or breast-feeding women
8. Patients with known hypersensitivity to Chinese hamster ovary cell products or other recombinant human, chimeric, or humanized antibodies.
9. Patients with active bleeding or pathologic conditions that carry a high risk of bleeding, (i.e. hereditary hemorrhagic telangiectasia).
10. Patients who are currently receiving anticoagulation treatment
11. Patients unwilling or unable to comply with the protocol

5.3. Patient Withdrawal Criteria

A patient should be withdrawn from study treatment if, in the opinion of the Investigator, it is medically necessary, or if it is the wish of the patient. If a patient does not return for a scheduled visit, every effort should be made to contact the patient. In any circumstance, every effort should be made to document patient outcome. Data to be collected at the end of study visit are described in the Schedule of Assessments (Table 4). Patients will be followed for at least 28 days following the last dose of TRC105 for adverse events. If the patient withdraws consent, no further evaluations should be performed, and no attempts should be made to collect additional data. In addition, patients will be withdrawn from treatment in the case of:

1. Modified RANO criteria defined disease progression. In cases where RANO cannot be applied, progression should be based on unequivocal evidence of progressive disease sufficient to require a change in therapy.

14. A need for surgery, radiation, or for other anticancer therapy not specified in the protocol.

15. TRC105 dose delay > 8 weeks.

16. Lost to follow-up or noncompliant.

17. Pregnancy. Pregnant patients should be followed for the duration of the pregnancy and the outcome of the pregnancy should be documented.

18. Arterial thrombosis of any grade (including cerebrovascular ischemia, cardiac ischemia/infarction, or peripheral or visceral arterial ischemia) or arterial or venous thrombosis of any grade requiring anticoagulation (including pulmonary embolism).

6. TREATMENT OF PATIENTS

6.1. Description of TRC105 Study Drug

TRC105 is a genetically engineered human/murine chimeric monoclonal antibody directed against human CD105 found on the surface of proliferating endothelial cells.

6.2. Composition of TRC105

TRC105 is an IgG1, kappa immunoglobulin containing murine light- and heavy-chain variable region sequences and human constant region sequences. TRC105 has an approximate molecular weight of 148 kDa.

6.3. TRC105 Dose Level

REDACTED

6.4. TRC105 Packaging and Labeling

REDACTED

6.5. TRC105 Storage and Shipping

REDACTED

6.6. TRC105 Preparation

REDACTED.

6.7. TRC105 Administration

REDACTED

6.8. TRC105 Study Drug Accountability

REDACTED.

6.9. TRC105 Study Drug Handling and Disposal

REDACTED

6.10. Bevacizumab Packaging

REDACTED

6.11. Bevacizumab Preparation

REDACTED

6.12. Bevacizumab Administration

REDACTED

6.12.1. Bevacizumab Dose Modification

REDACTED

6.13. Bevacizumab Drug Accountability

REDACTED

6.14. Bevacizumab Handling and Disposal

REDACTED

6.15. Concomitant Medications

REDACTED

6.16. TRC105 Study Drug Treatment Compliance

REDACTED

6.17. Patient Enrollment

REDACTED

7. ASSESSMENT OF EFFICACY

7.1. Response Assessment

Optimal guidelines for determining GBM response to therapy are not yet well established. The most accepted are the Macdonald criteria [64]. However, increasingly specific criteria are being developed by the Response Assessment in Neuro-Oncology (RANO) Group through the American Society of Clinical Oncology. Using these developing guidelines, we have designed response criteria defined in Table 8 . These are largely based on standardized response criteria using bi-dimensional measurements of the largest contrast-enhancing area [65]. However, it has been demonstrated that contrast-enhanced images can be altered by agents inhibiting angiogenesis with occasional progression of T2-weighted or FLAIR abnormality as well as clinical decline despite improvement in the contrast-enhancing signal [66, 67]. In light of this, FLAIR imaging and clinical status will be part of the response criteria in addition to the widely used Macdonald criteria. Hence, the largest cross-sectional area on the T1-weighted contrast-enhanced images will be selected and measured in 2 dimensions with linear measures on the baseline MRI axial sequence. In addition, the largest cross-sectional area of a contiguous hyperintense lesion on FLAIR sequences will be measured on the baseline MRI axial sequence. All subsequent scans will be compared against these baseline measures (for both CE and FLAIR). New foci of FLAIR signal abnormality will be recorded on each subsequent evaluation. Response will be scored based on a combination of imaging and clinical features as defined by the modified RANO Response Evaluation Criteria (Table 8).

Table 2: RANO Definitions of Tumor Response

Response	T1 Contrast Enhancement (CE)	FLAIR Images	Steroids	Neurologic Exam
**Complete Response (CR)**	No residual CE (complete disappearance of all enhancing measurable disease for at least 4 weeks; confirmatory MRI at 4 weeks is required to score as CR) and no new lesions.	Stable or reduced area of FLAIR signal abnormality	No steroids	Stable or improved from prior evaluation
**Partial Response (PR)**	>50% reduction in sum of products of the perpendicular diameters of all measureable enhancing lesions sustained for at least 4 weeks and no new lesions or progression of non-measurable lesions	Stable or reduced area of FLAIR signal abnormality	Stable or reduced glucocorticoids from baseline MRI	Stable or improved from prior evaluation
**Minor response (MR)**	>25% reduction in sum of products of the perpendicular diameters of all measureable enhancing lesions and no new lesions (confirmatory MRI at 4 weeks is required to score as PR)	Stable or reduced area of FLAIR signal abnormality	Stable or reduced glucocorticoids from baseline MRI	Stable or improved from prior evaluation
**Stable Disease (SD)**	<25% reduction in area of CE maintained for at least 4 weeks duration. Does not qualify for CR, PR or progression	Stable or reduced area of FLAIR signal abnormality	Stable or reduced glucocorticoids from baseline MRI	Stable or improved from prior evaluation

**Progressive Disease**	>25% in the sum of products of the perpendicular diameters of CE lesions; evidence of new lesion(s).	Measurable increase in the sum of products of the perpendicular diameters of FLAIR signal abnormality from the baseline scan or the scan representing the best response (if there was a response) following therapy and not attributable to other co-morbid events (seizure, radiation, injury, infection, ischemia, etc.) OR presence of a new focus of FLAIR signal abnormality that cannot be explained by any other pathologic process.	Stable or increased dose of glucocorticoids	Stable or worsening neurologic symptoms

Table 3: Modified RANO Response Evaluation Criteria

SPD	Non Measurable Lesions	New Lesion	FLAIR Images	Steriods	Neurologic Exam	Overall Response
No CE	Absent	No	Reduced	None	Improved	CR
No CE	Absent	No	Reduced	None	Stable	CR
No CE	Absent	No	Stable	None	Improved	CR
No CE	Absent	No	Stable	None	Stable	CR
> 50% Reduction	Not "Increased" and Not "Clear Progreesion"	No	Reduced	Reduced	Improved	PR
> 50% Reduction		No	Reduced	Reduced	Stable	PR
> 50% Reduction		No	Stable	Reduced	Improved	PR
> 50% Reduction		No	Stable	Reduced	Stable	PR
> 50% Reduction		No	Reduced	Stable	Improved	PR
> 50% Reduction		No	Reduced	Stable	Stable	PR
> 50% Reduction		No	Stable	Stable	Improved	PR
> 50% Reduction		No	Stable	Stable	Stable	PR
> 25% Reduction	Not "Increased" and Not "Clear Progreesion"	No	Reduced	Reduced	Improved	MR
> 25% Reduction		No	Reduced	Reduced	Stable	MR
> 25% Reduction		No	Stable	Reduced	Improved	MR
> 25% Reduction		No	Stable	Reduced	Stable	MR
> 25% Reduction		No	Reduced	Stable	Improved	MR
> 25% Reduction		No	Reduced	Stable	Stable	MR
> 25% Reduction		No	Stable	Stable	Improved	MR
> 25% Reduction		No	Stable	Stable	Stable	MR
< 25% Reduction	Not "Clear Progression"	No	Reduced	Reduced	Improved	SD
< 25% Reduction		No	Reduced	Reduced	Stable	SD
< 25% Reduction		No	Stable	Reduced	Improved	SD
< 25% Reduction		No	Stable	Reduced	Stable	SD
< 25% Reduction		No	Reduced	Stable	Improved	SD
< 25% Reduction		No	Reduced	Stable	Stable	SD
< 25% Reduction		No	Stable	Stable	Improved	SD
< 25% Reduction		No	Stable	Stable	Stable	SD
> 25% Increase	N/A	N/A	N/A	N/A	N/A	PD
N/A	N/A	Yes	N/A	N/A	N/A	PD
N/A	Clear Progression	N/A	N/A	N/A	N/A	PD
N/A	N/A	N/A	Measurable increase from baseline or best response **OR** new focus of FLAIR signal	N/A	N/A	PD

Radiological tumor assessments will be performed at screening, as outlined in the Schedule of Assessments (Table 4), and whenever disease progression is suspected. Another tumor assessment will be performed at the End of Study Visit if an assessment has not been performed within the prior 8 weeks. All patient files and radiological images must be available for CRF source verification.

8. ASSESSMENT OF SAFETY

8.1. Safety Parameters

Safety will be characterized in terms of the incidence, timing, severity (graded by the National Cancer Institute [NCI] Common Terminology Criteria for Adverse Events [CTCAE], Version 4.0), seriousness, and relatedness of adverse events and laboratory abnormalities. In addition, physical examination, vital signs, and Karnofsky performance status will be serially monitored. Laboratory safety analyses will be based on the local laboratory data, and will include hematology, serum chemistry (including liver, kidney and thyroid function), urinalysis, serum or urine pregnancy testing, and coagulation profile. Serum will also be assessed for immunogenicity to TRC105 (including HACA and HAMA titers). In addition, an ECG will be recorded at baseline and as clinically indicated throughout the study.

8.1.1. Laboratory Safety Assessments

Abnormal and clinically significant laboratory tests should be recorded as adverse events. To meet the definition of clinically significant, the test result generally requires a change in medical management (e.g. new medication, unplanned treatment, additional tests, etc.).

8.1.1.1. Hematology, Serum Chemistry, Coagulation, Pregnancy Test

Assessments will be performed at the time points indicated in the Schedule of Assessments (Table 4) and analyzed at local laboratories. Investigators may have additional blood tests performed for the purpose of planning treatment administration, or for following adverse events as clinically indicated.

- Hematology: CBC with differential and platelet count
- Coagulation: Prothrombin Time (PT) or International Normalized Ration (INR) will be assessed
- Serum Chemistry: Total bilirubin, alanine transaminase (ALT), aspartate transaminase (AST), alkaline phosphatase, total protein, albumin, lipase, sodium, potassium, bicarbonate, chloride, calcium, phosphorus, blood urea nitrogen, creatinine, glucose, and TSH
- Pregnancy Test: Serum or urine pregnancy tests will be performed locally on all female patients of childbearing potential. Patients must be surgically sterile (i.e.: hysterectomy) or be postmenopausal, or must agree to use effective contraception during the study and for 3 months following last dose of TRC105. The definition of effective contraception will be based on the judgment of the Principal Investigator or a designated associate.

8.1.1.2. Urinalysis

Urinalysis will be performed at time points indicated in the Schedule of Assessments (Table 4) and analyzed by local laboratories. Microscopic analysis and/or urine protein-creatinine ratio (UPCR) should be performed as clinically indicated.

8.1.1.3. Physical Examination

A physical examination including, but not limited to, general appearance, head, eyes, ears, nose, throat, neck, heart, chest, abdomen, musculoskeletal, extremities, skin, lymph nodes, neurological genitourinary (as appropriate), and rectal (as appropriate) will be assessed at time points indicated within the Schedule of Assessments (Table 4). The physical examination will include examination of known and suspected sites of disease.

8.1.1.4. Vital Signs

Heart rate, temperature, blood pressure, respiratory rate and weight will be assessed at time points indicated within the Schedule of Assessments (Table 4). Heart rate, temperature, blood pressure, and respiratory rate will also be assessed during TRC105 infusions as described in Section 4.1.2.2 and the footnotes of the Table 4 Schedule of Assessments.

8.1.1.5. Performance Status

The Karnofsky scale will be used to assess performance status at Screening.

8.1.1.6. ECG

A single 12-lead (with a 10–second rhythm strip) tracing will be used for all ECGs. It is preferable that the machine used has a capacity to calculate standard intervals automatically. ECG will be performed according to the Schedule of Assessments (Table 4) and as clinically indicated throughout the study.

8.2. Adverse Events

All observed or volunteered adverse events regardless of suspected causal relationship to TRC105 study drug will be reported as described below.

8.2.1. Definition of Adverse Event

An adverse event is any untoward medical occurrence in a trial patient who is administered a drug or biologic (medicinal product); the event may or may not have a causal relationship with the medicinal product. Examples of adverse events include, but are not limited to the following:

- Clinically significant symptoms and signs including:
    - Worsening of signs and symptoms of the malignancy under trial (disease progression without worsening of signs and symptoms assessed by measurement of malignant lesions on radiographs or other methods should not be reported as adverse events).
    - Signs and symptoms resulting from drug overdose, abuse, misuse, withdrawal, sensitivity, dependency, interaction or toxicity.
    - All possibly related and unrelated illnesses, including the worsening of a preexisting illness.
    - Injury or accidents. Note that if a medical condition is known to have caused the injury or accident (hip fracture from a fall secondary to dizziness), the medical

condition (dizziness) and the outcome of the accident (hip fracture from a fall) should be reported as 2 separate adverse events.

  - Symptoms or signs resulting from exposure *in utero*.

- Abnormalities in physiological testing or physical examination findings that require clinical intervention or further investigation (beyond ordering a repeat confirmatory test).
- Laboratory abnormalities that meet any of the following (Note: merely repeating an abnormal test, in the absence of any of the below conditions, does not constitute an adverse event. Any abnormal test result that is determined to be an error does not require reporting as an adverse event.):
  - Test result that is associated with accompanying symptoms
  - Test result that requires additional diagnostic testing or medical/surgical intervention
  - Test result that leads to a change in TRC105 study drug dosing outside of protocol-stipulated dose adjustments or discontinuation from the trial, significant additional concomitant drug treatment, or other therapy
  - Test result that is considered to be an adverse event by the Investigator or TRACON

8.2.2. Serious Adverse Events

An adverse event that meets one or more of the following criteria/outcomes is classified as serious:

- Results in death
- Is life-threatening (at immediate risk of death)
- Requires in patient hospitalization or prolongation of existing hospitalization
- Results in persistent or significant disability/incapacity
- Results in congenital anomaly/birth defect
- Other important medical events that may not result in death, be life-threatening, or require hospitalization may be considered serious when, based upon appropriate medical judgment, they may jeopardize the patient or may require medical or surgical intervention to prevent one of the outcomes listed above. Examples of such events are intensive treatment in an emergency room for allergic bronchospasm; blood dyscrasias or convulsions that do not result in hospitalization; or the development of drug dependence or drug abuse.

Serious also includes any other event that the Investigator or sponsor judges to be serious, or which is defined as serious by the HRA in the country in which the event occurred.

Progression of the malignancy under study (including signs and symptoms of progression) should not be reported as SAEs unless the outcome is fatal during the trial or within the safety reporting period. Hospitalizations due to signs and symptoms of disease progression should not

be reported as SAEs. If the malignancy has a fatal outcome during the trial or within the safety reporting period, then the event leading to death must be recorded as an adverse event and as an SAE with CTC grade 5.

The onset date of an SAE is defined as the date on which the event initially met serious criteria (e.g., the date of admission to a hospital). The end date is the date on which the event no longer met serious criteria (e.g., the date the patient was discharged from a hospital).

8.2.2.1. Hospitalization

Adverse events associated with in-patient hospitalization, or prolongation of an existing hospitalization, are considered serious. Any initial admission, even if the duration is less than 24 hours is considered serious. In addition, any transfer within the hospital to an acute/intensive care unit is considered serious (e.g., transfer from the psychiatric wing to a medical floor or transfer from a medical floor to a coronary care unit). However, the following hospitalizations **should not** be considered serious:

- Rehabilitation facility admission
- Hospice facility admission
- Respite care
- Skilled nursing facility admission
- Nursing home admission
- Emergency room visit
- Same day surgery
- Hospitalization or prolongation of hospitalization in the absence of precipitating clinical adverse events as follows:
    - Admission for treatment of preexisting condition not associated with the development of a new adverse event or with a worsening of the preexisting condition
    - Social admission
    - Administrative admission (e.g. for yearly physical exam)
    - Protocol-specified admission during a clinical trial
    - Optional admission not associated with a precipitating clinical adverse event (e.g. for elective cosmetic surgery)
    - Preplanned treatments or surgical procedures
    - Admission exclusively for the administration of blood products

Diagnostic and therapeutic noninvasive and invasive procedures, such as surgery, should not be reported as adverse events. The medical condition for which the procedure was performed **should** be reported if it meets the definition of an adverse event (e.g. acute appendicitis that

begins during the adverse event reporting period should be reported as an adverse event and the appendectomy should be recorded as a concomitant treatment).

8.3. Reporting Adverse Events

8.3.1. Eliciting Adverse Event Information

The Investigator is to report all directly observed adverse events and all adverse events spontaneously reported by the trial patient using concise medical terminology. In addition, each trial patient will be questioned about adverse events at each clinic visit following initiation of treatment. The question asked will be, "Since your last clinic visit have you had any health problems?"

8.3.2. Adverse Event Reporting Period

Safety information for each patient will be collected from the date of informed consent. Adverse events occurring prior to the initiation of the study treatment will be considered "baseline-emergent adverse events" and will be recorded on corresponding case report forms. The adverse event reporting period for this trial begins when the patient has taken the first dose of TRC105 study drug and ends 28 days following the last dose of TRC105 as outlined in the Schedule of Assessments.

All adverse events that occur in trial patients during the adverse event reporting period specified in the protocol must be reported to TRACON, whether or not the event is considered study treatment-related. In addition, any known untoward event that occurs beyond the adverse event reporting period that the Investigator assesses as possibly related to the investigational medication/product should also be reported as an adverse event.

8.3.3. Reporting Requirements

Each adverse event is to be classified by the Investigator as SERIOUS or NONSERIOUS. This classification of the gravity of the event determines the reporting procedures to be followed. If an SAE occurs, reporting will follow local and international regulations, as appropriate.

REDACTED

Following notification, the Investigator will report the SAE via the AE CRF via the data management system. The initial AE CRF is to be updated with followed more detailed adverse event information within **5 calendar days** of the event.

In the rare event that the Investigator is not immediately aware of an SAE (for example, if the study subject seeks urgent medical attention elsewhere), the Investigator is to notify the Sponsor immediately upon learning about the event and document his/her first awareness.

Each SAE should be followed until resolution, or until such time as the Investigator determines its cause or determines that it has become stable. Information pertaining to follow-up of SAEs should also be sent to the TRACON Pharmaceuticals Inc.

Serious adverse events that are unexpected and associated with use of the study medication will be reported to the US Food and Drug Administration (FDA) and all participating clinical sites by TRACON via MedWatch forms. For events which are fatal or life-threatening, unexpected, and

associated with use of the investigational product, a 7-Day Alert Report will be submitted to the FDA within 7 calendar days of receipt of the SAE information. For all other events that are serious, unexpected, and associated with use of the investigational product, a written report will be made no more than 15 calendar days from the date TRACON learns of the event. Participating clinical sites will be notified of these events in parallel.

All adverse events, including SAEs, are to be reported on the adverse event CRFs.

8.3.4. Recording Adverse Events in the Case Report Forms

REDACTED

8.3.5. Grading of Adverse Event Severity

To report adverse events on the CRFs, the Investigator will use the severity grading as described in NCI CTCAE (Version 4.0).

Every effort should be made by the Investigator to assess the adverse event according to CTCAE criteria. If the Investigator is unable to assess severity because the term is not described in NCI (Version 4.0), severity of MILD, MODERATE, SEVERE, LIFE-THREATENING, or FATAL may be used to describe the maximum intensity of the adverse event. For purposes of consistency, these intensity grades are defined as follows:

Table 4: Adverse Event Grading

Grade	Non-CTCAE Severity	Definition
1	Mild	Does not interfere with patient's usual function
2	Moderate	Interferes to some extent with patient's usual function
3	Severe	Interferes significantly with patient's usual function
4	Life-Threatening	Results in immediate risk of patient's death
5	Fatal	Results in patient's death

Note the distinction between the severity and the seriousness of an adverse event. A severe event is not necessarily a serious event. For example, a headache may be severe (interferes significantly with patient's usual function) but would not be classified as serious unless it met one of the criteria for serious events.

8.3.6. Relationship to TRC105 Study Drug

In this study, TRC105 study drug is given in combination with bevacizumab. The relationship of an adverse event to TRC105 study drug should be classified by the Investigator using the following guidelines:

- Suspected Adverse Reaction: There is a reasonable possibility that TRC105 caused the adverse event (i.e.: there is evidence to suggest a causal relationship between TRC105 and adverse event).
- Not Related: There is no reasonable possibility that the adverse event is associated with TRC105 study drug.

AE's related to TRC105 study drug or bevacizumab are considered Adverse Drug Reactions (ADR).

8.3.7. Expectedness

All adverse events and adverse drug reactions are reaction considered "unexpected" if it not listed in the investigator brochure or not listed at the specificity or severity that has been observed. For example, under this definition, hepatic necrosis would be unexpected (by virtue of greater severity) if the investigator brochure referred only to elevated hepatic enzymes or hepatitis. Similarly, cerebral thromboembolism and cerebral vasculitis would be unexpected (by virtue of greater specificity) if the investigator brochure listed only cerebral vascular accidents. "Unexpected," as used in this definition, also refers to adverse events or suspected adverse reactions that are mentioned in the investigator brochure as occurring with a class of drugs or as anticipated from the pharmacological properties of the drug, but are not specifically mentioned as occurring with the particular drug under investigation.

8.3.8. Exposure *in Utero*

An exposure *in utero* (EIU) occurs if:

- A female becomes, or is found to be, pregnant either while receiving or having been directly exposed to (e.g., environmental exposure) the investigational product, or the female becomes, or is found to be, pregnant after discontinuing and/or being directly exposed to the investigational product (maternal exposure)
- A male has been exposed, either due to treatment or environmental, to the investigational product prior to or around the time of conception and/or is exposed during the partner's pregnancy (paternal exposure)

If any trial patient becomes or is found to be pregnant during the study or within 28 days of discontinuing the investigational medication/product, the Investigator must report the information to TRACON, or designee via the Pregnancy Notification Report Form. This must be done irrespective of whether an adverse event has occurred and within 24 hours of awareness of the pregnancy. The information submitted should include the anticipated date of delivery.

The Investigator will follow the patient until completion of the pregnancy or until pregnancy termination (i.e., induced abortion) and then notify TRACON, or its designee, of the outcome within 5 days or as specified below. The Investigator will provide this information as a follow-up to the initial report. The reason(s) for an induced abortion must be specified.

The Investigator should follow procedures for reporting an SAE if pregnancy outcome meets criteria for an SAE (i.e., spontaneous abortion, stillbirth, neonatal death, or congenital anomaly [including that in an aborted fetus]).

In the case of a live birth, the "normality" of the newborn can be assessed at the time of birth and the Pregnancy Outcome Report Form should be completed (i.e., no minimum follow-up period of a presumably normal infant must pass before a Pregnancy Outcome Report Form can be completed). The "normality" of an aborted fetus can be assessed by gross visual inspection unless pre-abortion laboratory findings are suggestive of a congenital anomaly.

Additional information about pregnancy outcomes that are classified as SAEs follows:

- "Spontaneous abortion" includes miscarriage and missed abortion.
- All neonatal deaths that occur within 1 month of birth should be reported, without regard to causality, as SAEs. In addition, any infant death after 1 month that the Investigator assesses as possibly related to the *in utero* exposure to the investigational medication should also be reported.

8.3.9. Follow-up of Unresolved Adverse Events

All adverse events should be followed until they are resolved or the Investigator assesses them as chronic or stable. Any increase or decrease in adverse event grade should be recorded as a new adverse event.

All serious and those non-serious events assessed by the Investigator as possibly related to the investigational medication/product should continue to be followed even after the patient's participation in the trial is over. Such events should be followed until they resolve or until the Investigator assesses them as "chronic" or "stable." The event should also be documented on the adverse event CRF.

8.4. Safety Monitoring

REDACTED

9. OTHER LABORATORY ASSESSMENTS

9.1. Pharmacokinetics

REDACTED

9.1.1. Predose TRC105 Concentration

REDACTED.

9.1.2. End of Infusion TRC105 Concentrations

REDACTED

9.2. TRC105 Immunogenicity

REDACTED

9.3. Protein Biomarkers

REDACTED.

10. STATISTICS

A one stage accrual design with an accrual goal of 22 evaluable patients will be employed in order to test the hypothesis that TRC105 + bevacizumab can increase the overall survival from 4 months to 7 months.

Assuming an approximately 10 % exclusion rate an additional 4 patients may be entered to replace ineligible patients or patients who withdraw consent prior to receiving study treatment. Assuming overall survival follows an exponential distribution, accrual takes approximately 12 months, and that there is 12 months of additional follow-up once accrual has been completed, there will be 81% power to detect the specified difference using a two-sided test with 10 % type I error.

A number of potential biomarkers are being assessed, and therefore to simplify the sample size discussion calculations are expressed in terms of standard deviations of the biomarker under study. Assuming 12-14 patients have correlative data there will be statistical power >80% to detect moderate to large treatment effects on the biomarkers and differences in them between specified patient groups (e.g. responders versus non-responders). That is, with 12-14 patients there will be ≥85% power to detect changes in a biomarker following treatment ≥1.0 standard deviation, and ≥84% power to detect differences ≥2.5 standard deviations between any two groups of patients even if only 1/3 of patients are in one of the groups. As an example, if the variability of the relative change in biomarker following treatment is as much as 50% there will be ≥85% power to detect a mean decrease ≥50%. Calculations are based on 2-sided Wilcoxon signed rank and rank sum tests with 5% Type I errors, respectively. The data will be analyzed primarily using non-parametric methods such as the Wilcoxon signed-rank test (for paired data such as pre- versus post-treatment changes) and rank sum test (to assess differences between groups of patients). Multivariable models such as linear and logistic regression and will be used to assess multiple factors. All tests of statistical significance will be two-sided and no adjustment will be made for multiple comparisons.

10.1. Definition of Analyzed Study Populations

Only those patients who are deemed "ineligible" or who receive no therapy will be eliminated from the analysis. All patients who receive any therapy will be evaluated for both treatment efficacy and toxicity. .

10.2. Data Analysis

Descriptive statistics (such as means, medians, standard deviations and ranges for continuous data and percentages for categorical data) will be used to summarize patient characteristics, treatment administration/compliance, immunogenicity (HAMA and HACA), efficacy, pharmacokinetic parameters and protein biomarkers. Data will also be displayed graphically, where appropriate.

10.2.1. Analysis of Efficacy and Safety

The primary efficacy analysis will calculate the proportion of patients who are progression free at 6 months and the proportion of patients who have objective tumor response (complete or partial) by modified RANO criteria. Overall survival and progression-free survival will be presented as Kaplan-Meier plots and estimates of the median time until death or the earlier of documented progression or death.

For a given event, the number and proportion of patients reporting it will be tabulated according to the worst severity experienced. Severity will be graded per NCI (Version 4.0). Individual tables will be constructed for (a) all reported adverse events, (b) adverse events reported as treatment related, and (c) serious adverse events.

10.2.2. Analysis of Protein Biomarkers

Angiogenic protein biomarker data for each patient who received at least one dose of study drug will be listed.

10.2.3. Analysis of Pharmacokinetics

Peak and trough serum TRC105 concentrations will be measured using validated ELISA methods.

10.2.4. Analysis of Immunogenicity

HAMA and HACA concentrations will be measured using validated ELISA methods. HAMA and HACA concentrations will be evaluated in the context of pharmacokinetic parameters and AE profiles.

11. DIRECT ACCESS TO SOURCE DATA/DOCUMENTS

All data entered on CRFs/eCRFs must be verifiable within the patients' source documents (written or electronic record). The Investigator/institution guarantees TRACON representatives and appropriate regulatory authorities direct access to the original source records for the duration of the agreed study record retention period. Printouts of source records that are electronically obtained and stored will not be acceptable for audit/inspection unless provided as certified exact copies and the data remains as meaningful and useful as in its original electronic state.

Legally protected subject identification and other personal health information must be securely stored with limited access by the participating institutions. Unless secure provisions are established by the institution to allow TRACON (or designee) to perform remote monitoring of electronic source records, TRACON (or designee) will review source records/data on site and will not remove any such protected health information.

12. QUALITY CONTROL AND QUALITY ASSURANCE

Monitoring visits to clinical investigator sites will be made by TRACON or its representatives periodically during the trial to ensure that GCPs and all aspects of the protocol are being followed.

The trial site will also be subject to possible inspection by the institutional review board (IRB) or independent ethics committee (IEC) or other appropriate regulatory authority. The trial site is also subject to quality assurance (QA) audits performed by TRACON or its representatives.

It is important that the Investigator(s) and their relevant personnel are available during the monitoring visits, audits, and inspections and that sufficient attention, time, and support is devoted to the process.

TRACON and its representatives will be governed by applicable regulations, good clinical practice standards, and internal SOPs for the conduct of monitoring visits and QA audits.

13. ETHICS

13.1. Institutional Review Board (IRB)/Independent Ethics Committee (IEC)

It is the responsibility of the Investigator to have approval of the trial protocol, protocol amendments, informed consent forms, and advertisements from the IRB/IEC before potential patients are consented for participation on the trial. All correspondence and other evidence of appropriate and timely communications with the IRB/IEC should be retained in the Investigator/site files. Copies of all IRB/IEC approvals should also be forwarded to TRACON.

The only circumstance in which an amendment may be initiated prior to IRB/IEC approval is where the change is necessary to eliminate apparent immediate hazards to the patients. In that event, the Investigator must notify the IRB/IEC and TRACON in writing within 5 business days after the implementation.

13.2. Ethical Conduct of the Study

The trial will be performed in accordance with the protocol, applicable local regulatory requirements and laws, and the International Conference on Harmonization Guideline on Good Clinical Practice, which supports the application of ethical principles that have their origin in the the Declaration of Helsinki (see ICH E6, §2.1).

13.3. Written Informed Consent

The informed consent form language must be agreed upon by TRACON and the IRB/IEC and must be in compliance with ICH GCP, local regulatory requirements, and legal requirements. The informed consent information must not be changed without prior approval by TRACON and the IRB/IEC. The informed consent form used in this trial, and any changes made during the course of the trial, must be approved by both the IRB/IEC and TRACON, or designee, before use.

It is the responsibility of the Investigator to give each patient full and adequate verbal and written information regarding the objective and procedures of the trial and the possible risks involved. This information must be provided to the patient prior to undertaking any trial-related procedure.

Patients must be informed about their right to withdraw from the trial at any time. Furthermore, it is the responsibility of the Investigator to ensure all subjects are appropriately informed before obtaining their signed and dated consent. Signatures from the investigator conducting the informed consent discussion should also be obtained prior to undertaking any trial-related procedure. Consent by a legally authorized representative is not permitted. Should an impartial witness be needed, ICHE6 requirements for impartial witnesses will apply. The Investigator will retain the original of each patient's signed consent form in the Investigator/site files.

13.4. Patient Compensation

Patients will not be compensated for participation in this trial; this will be outlined in the patient informed consent form.

14. DATA HANDLING AND RECORDKEEPING

14.1. Inspection of Records

CRF's are required and should be completed for each patient who receives treatment with TRC105. Screen failure CRF's will not be collected unless the patient experiences an SAE prior to receiving his/her first dose of TRC105, in which case demography and AE CRF's should be completed. Nevertheless, records of potential patients identified and screened shall be retained.

The completed original CRFs are the sole property of TRACON and should not be made available in any form to third parties without written permission from TRACON (except for authorized representatives of the HRA and in accordance with HIPAA regulations). It is the Investigator's responsibility to ensure completion and to review and approve all CRF data. The investigator will sign off on his/her data per patient. These signatures serve to attest that the investigator has reviewed and approved the information contained on the case report forms and that the information is complete, accurate, and true. At all times, the Investigator has final personal responsibility for the accuracy and authenticity of all clinical and laboratory data entered on the CRFs.

The use of electronic CRFs (eCRFs) to capture study data using automated computerized data capture systems does not change the principles and requirements for collecting study data. The investigator still retains final personal responsibility for eCRF data and any associated data pertaining to it (e.g. metadata including any record of change to the originally recorded data).

The investigator's signed approval of the eCRF data serves to attest that the electronic data and all of its associated metadata (including changes) has been reviewed and accepted as complete, accurate, and true for each patient in the study.

All CRF/eCRF data must be verifiable in the patient's source records by TRACON or its designee. TRACON will review CRF data as compared to source records in an attempt to identify missing and spurious data and notify the investigator of findings so that proper corrections can be made. TRACON representatives (monitors and auditors), and regulatory inspectors shall have direct access to the original source records in its original recorded format: electronic or hardcopy.

TRACON (or its designee) will perform all data management functions associated with the study. Data will be captured electronically. Automated data verification ("edit checks") will be used to ensure that the data are logical and consistent. Any inconsistencies will be queried for clarification or correction as appropriate by the clinical site.

14.2. Retention of Records

To allow for appropriate evaluations and/or audits by regulatory authorities or TRACON, the Investigator agrees to keep records, including the identity of all participating patients (sufficient information to link records, CRFs and hospital records), all original signed informed consent forms, copies of all CRFs, source documents, and detailed records of treatment disposition. The Investigator should

retain these records according to local regulations or as specified in the Clinical Trial Agreement, whichever is longer.

If the Investigator relocates, retires, or for any reason withdraws from the study, then TRACON should be prospectively notified. The study records must be transferred to an acceptable designee, such as another Investigator, another institution, or to TRACON. The Investigator must inform TRACON of any such transfer of responsibilities and properly identify the person or institution assuming the responsibility. The responsible investigator/institution must obtain TRACON's written permission before disposing of any records.

15. DEFINITION OF END OF TRIAL

15.1. End of Trial in all Participating Countries

End of trial in all participating countries is defined as the time at which all patients enrolled in the study have completed treatment on study.

15.2. TRACON Discontinuation Criteria

Premature termination of this trial may occur because of a regulatory authority decision, change in opinion of the IRB/IEC, drug safety problems, or at the discretion of TRACON. In addition, TRACON retains the right to discontinue development of TRC105 at any time. TRACON reserves the right to discontinue the trial prior to inclusion of the intended number of patients, but intends only to exercise this right for valid scientific or administrative reasons. If a trial is prematurely terminated or discontinued, TRACON will promptly notify the Investigator. After notification, the Investigator must contact all participating patients within a 28 day time period. As directed by TRACON, all trial materials must be collected and all CRF data must be completed to the greatest extent possible.

16. PUBLICATION OF TRIAL RESULTS

Publication of trial results is discussed in the Clinical Trial Agreement.

17. FINANCING AND INSURANCE

Financing and Insurance are discussed in the Clinical Trial Agreement.

18. INVESTIGATOR PROTOCOL AGREEMENT: 105GM201

I understand that all information concerning this study supplied to me by TRACON Pharmaceuticals, Inc. is confidential information. I have read this protocol and agree to conduct the study according to all applicable regulations, Good Clinical Practice Guidelines and in accordance with the Clinical Trial Agreement.

I understand that this protocol and all amendments must be submitted to the appropriate IRB/IEC.

Investigator Name (PLEASE PRINT): ____

Signature: ____Date: _______________

Please sign and return this agreement to:

TRACON Pharmaceuticals, Inc.
Attn: Clinical Operations
8910 University Center Lane, Suite 700
San Diego, CA 92122

Please keep a copy for your records.

19. REFERENCES

1. Stupp, R., et al., *Radiotherapy plus concomitant and adjuvant temozolomide for glioblastoma.* N Engl J Med, 2005. **352**(10): p. 987-96.

2. Carmeliet, P., *Angiogenesis in health and disease.* Nat Med, 2003. **9**(6): p. 653-60.

3. Folkman, J. and Y. Shing, *Angiogenesis.* J Biol Chem, 1992. **267**(16): p. 10931-4.

4. Sandler, A., et al., *Paclitaxel-carboplatin alone or with bevacizumab for non-small-cell lung cancer.* N Engl J Med, 2006. **355**(24): p. 2542-50.

5. Hurwitz, H., et al., *Bevacizumab plus irinotecan, fluorouracil, and leucovorin for metastatic colorectal cancer.* N Engl J Med, 2004. **350**(23): p. 2335-42.

6. Miller, K.D., et al., *Randomized phase III trial of capecitabine compared with bevacizumab plus capecitabine in patients with previously treated metastatic breast cancer.* J Clin Oncol, 2005. **23**(4): p. 792-9.

7. Friedman, H.S., et al., *Bevacizumab alone and in combination with irinotecan in recurrent glioblastoma.* J Clin Oncol, 2009. **27**(28): p. 4733-40.

8. Escudier, B., et al., *Phase III trial of bevacizumab plus interferon alfa-2a in patients with metastatic renal cell carcinoma (AVOREN): final analysis of overall survival.* J Clin Oncol, 2010. **28**(13): p. 2144-50.

9. Rini, B.I., *Vascular endothelial growth factor-targeted therapy in metastatic renal cell carcinoma.* Cancer, 2009. **115**(10 Suppl): p. 2306-12.

10. Motzer, R.J., et al., *Sunitinib versus interferon alfa in metastatic renal-cell carcinoma.* N Engl J Med, 2007. **356**(2): p. 115-24.

11. Llovet, J.M., et al., *Sorafenib in advanced hepatocellular carcinoma.* N Engl J Med, 2008. **359**(4): p. 378-90.

12. Maxwell, M., et al., *Expression of angiogenic growth factor genes in primary human astrocytomas may contribute to their growth and progression.* Cancer Res, 1991. **51**(4): p. 1345-51.

13. Takahashi, J.A., et al., *Correlation of basic fibroblast growth factor expression levels with the degree of malignancy and vascularity in human gliomas.* J Neurosurg, 1992. **76**(5): p. 792-8.

14. Stefanik, D.F., et al., *Monoclonal antibodies to vascular endothelial growth factor (VEGF) and the VEGF receptor, FLT-1, inhibit the growth of C6 glioma in a mouse xenograft.* J Neurooncol, 2001. **55**(2): p. 91-100.

15. Kerr, C., *Bevacizumab and chemotherapy improves survival in NSCLC.* Lancet Oncol, 2005. **6**(5): p. 266.

16. Kreisl, T.N., et al., *Phase II trial of single-agent bevacizumab followed by bevacizumab plus irinotecan at tumor progression in recurrent glioblastoma.* J Clin Oncol, 2009. **27**(5): p. 740-5.

17. *Avastin Package Insert*. Available from: http://www.gene.com/gene/products/information/pdf/avastin-prescribing.pdf.

18. Iwamoto, F.M., et al., *Patterns of relapse and prognosis after bevacizumab failure in recurrent glioblastoma.* Neurology, 2009. **73**(15): p. 1200-6.

19. Norden AD, Y.G., Setayesh K, et al. , *Bevacizumab for recurrent malignant gliomas: efficacy, toxicity, and patterns of recurrence.* Neurology, 2008. **70**: p. 779-787.

20. Quant EC, N.A., Drappatz J, et al. , *Role of a second chemotherapy in recurrent malignant glioma patients who progress on bevacizumab.* . Neuro Oncol, 2009. **11**: p. 550-555.

21. Reardon DA, H.J.n., Peters KB et al. , *bevacizumab continuation beyond initial bevacizumab progression among recurrent glioblastoma patients.* Br j cancer. , 2012. **107(9)**(Oct23): p. 1481-1487.

22. Haruta, Y. and B.K. Seon, *Distinct human leukemia-associated cell surface glycoprotein GP160 defined by monoclonal antibody SN6.* Proc Natl Acad Sci U S A, 1986. **83**(20): p. 7898-902.

23. Gougos, A. and M. Letarte, *Identification of a human endothelial cell antigen with monoclonal antibody 44G4 produced against a pre-B leukemic cell line.* J Immunol, 1988. **141**(6): p. 1925-33.

24. Seon, B.K., *Similarity of TALLA-1, a T-cell acute lymphoblastic leukemia antigen, to GP37.* Int J Cancer, 1995. **63**(3): p. 474.

25. Rokhlin, O.W., et al., *Differential expression of endoglin on fetal and adult hematopoietic cells in human bone marrow.* J Immunol, 1995. **154**(9): p. 4456-65.

26. Cheifetz, S., et al., *Endoglin is a component of the transforming growth factor-beta receptor system in human endothelial cells.* J Biol Chem, 1992. **267**(27): p. 19027-30.

27. Li, D.Y., et al., *Defective angiogenesis in mice lacking endoglin.* Science, 1999. **284**(5419): p. 1534-7.

28. Burrows, F.J., et al., *Up-regulation of endoglin on vascular endothelial cells in human solid tumors: implications for diagnosis and therapy.* Clin Cancer Res, 1995. **1**(12): p. 1623-34.

29. Seon, B.K., et al., *Long-lasting complete inhibition of human solid tumors in SCID mice by targeting endothelial cells of tumor vasculature with antihuman endoglin immunotoxin.* Clin Cancer Res, 1997. **3**(7): p. 1031-44.

30. Seon, B.K., *Expression of endoglin (CD105) in tumor blood vessels.* Int J Cancer, 2002. **99**(2): p. 310-1; author reply 312.

31. Horsman, M.R. and D.W. Siemann, *Pathophysiologic effects of vascular-targeting agents and the implications for combination with conventional therapies.* Cancer Res, 2006. **66**(24): p. 11520-39.

32. Matsuno, F., et al., *Induction of lasting complete regression of preformed distinct solid tumors by targeting the tumor vasculature using two new anti-endoglin monoclonal antibodies.* Clin Cancer Res, 1999. **5**(2): p. 371-82.

33. Takahashi, N., et al., *Antiangiogenic therapy of established tumors in human skin/severe combined immunodeficiency mouse chimeras by anti-endoglin (CD105) monoclonal antibodies, and synergy between anti-endoglin antibody and cyclophosphamide.* Cancer Res, 2001. **61**(21): p. 7846-54.

34. Tsujie, M., et al., *Anti-tumor activity of an anti-endoglin monoclonal antibody is enhanced in immunocompetent mice.* Int J Cancer, 2008. **122**(10): p. 2266-73.

35. Uneda, S., H. Toi, and B.K. Seon, *Anti-endoglin monoclonal antibodies are effective for suppressing metastasis and the primary tumors by targeting tumor vasculature.* Int J Cancer, 2009. **125**: p. 1446.

36. Barbara, N.P., J.L. Wrana, and M. Letarte, *Endoglin is an accessory protein that interacts with the signaling receptor complex of multiple members of the transforming growth factor-beta superfamily.* J Biol Chem, 1999. **274**(2): p. 584-94.

37. She, X., et al., *Synergy between anti-endoglin (CD105) monoclonal antibodies and TGF-beta in suppression of growth of human endothelial cells.* Int J Cancer, 2004. **108**(2): p. 251-7.

38. Guo, B., et al., *CD105 inhibits transforming growth factor-beta-Smad3 signalling.* Anticancer Res, 2004. **24**(3a): p. 1337-45.

39. Sanchez-Elsner, T., et al., *Endoglin expression is regulated by transcriptional cooperation between the hypoxia and transforming growth factor-beta pathways.* J Biol Chem, 2002. **277**(46): p. 43799-808.

40. van Laake, L.W., et al., *Endoglin has a crucial role in blood cell-mediated vascular repair.* Circulation, 2006. **114**(21): p. 2288-97.

41. Lenato, G.M. and G. Guanti, *Hereditary Haemorrhagic Telangiectasia (HHT): genetic and molecular aspects.* Curr Pharm Des, 2006. **12**(10): p. 1173-93.

42. Sabba, C., et al., *Life expectancy in patients with hereditary haemorrhagic telangiectasia.* Qjm, 2006. **99**(5): p. 327-34.

43. Bockhorn, M., et al., *Differential vascular and transcriptional responses to anti-vascular endothelial growth factor antibody in orthotopic human pancreatic cancer xenografts.* Clin Cancer Res, 2003. **9**(11): p. 4221-6.

44. Davis, D.W., et al., *Regional effects of an antivascular endothelial growth factor receptor monoclonal antibody on receptor phosphorylation and apoptosis in human 253J B-V bladder cancer xenografts.* Cancer Res, 2004. **64**(13): p. 4601-10.

45. Yao, Y., et al., *Prognostic significance of microvessel density determined by an anti-CD105/endoglin monoclonal antibody in astrocytic tumors: comparison with an anti-CD31 monoclonal antibody.* Neuropathology, 2005. **25**(3): p. 201-6.

46. Kumar, S., et al., *Breast carcinoma: vascular density determined using CD105 antibody correlates with tumor prognosis.* Cancer Res, 1999. **59**(4): p. 856-61.

47. Vo, M.N., et al., *Elevated plasma endoglin (CD105) predicts decreased response and survival in a metastatic breast cancer trial of hormone therapy.* Breast Cancer Res Treat, 2008.

48. Rubatt, J.M., et al., *Independent prognostic relevance of microvessel density in advanced epithelial ovarian cancer and associations between CD31, CD105, p53 status, and angiogenic marker expression: A Gynecologic Oncology Group study.* Gynecol Oncol, 2009. **112**(3): p. 469-74.

49. Taskiran, C., et al., *The prognostic value of endoglin (CD105) expression in ovarian carcinoma.* Int J Gynecol Cancer, 2006. **16**(5): p. 1789-93.

50. Tanaka, F., et al., *Evaluation of angiogenesis in non-small cell lung cancer: comparison between anti-CD34 antibody and anti-CD105 antibody.* Clin Cancer Res, 2001. **7**(11): p. 3410-5.

51. El-Gohary, Y.M., et al., *Endoglin (CD105) and vascular endothelial growth factor as prognostic markers in prostatic adenocarcinoma.* Am J Clin Pathol, 2007. **127**(4): p. 572-9.

52. Svatek, R.S., et al., *Preoperative plasma endoglin levels predict biochemical progression after radical prostatectomy.* Clin Cancer Res, 2008. **14**(11): p. 3362-6.

53. Li, C., et al., *Both high intratumoral microvessel density determined using CD105 antibody and elevated plasma levels of CD105 in colorectal cancer patients correlate with poor prognosis.* Br J Cancer, 2003. **88**(9): p. 1424-31.

54. Romani, A.A., et al., *The risk of developing metastatic disease in colorectal cancer is related to CD105-positive vessel count.* J Surg Oncol, 2006. **93**(6): p. 446-55.

55. Ding, S., et al., *Comparative evaluation of microvessel density determined by CD34 or CD105 in benign and malignant gastric lesions.* Hum Pathol, 2006. **37**(7): p. 861-6.

56. Erdem, O., et al., *CD105 expression is an independent predictor of survival in patients with endometrial cancer.* Gynecol Oncol, 2006. **103**(3): p. 1007-11.

57. Yang, L.Y., et al., *Correlation between CD105 expression and postoperative recurrence and metastasis of hepatocellular carcinoma.* BMC Cancer, 2006. **6**: p. 110.

58. Saad, R.S., et al., *Endoglin (CD105) and vascular endothelial growth factor as prognostic markers in esophageal adenocarcinoma.* Hum Pathol, 2005. **36**(9): p. 955-61.

59. Kyzas, P.A., N.J. Agnantis, and D. Stefanou, *Endoglin (CD105) as a prognostic factor in head and neck squamous cell carcinoma.* Virchows Arch, 2006. **448**(6): p. 768-75.

60. Marioni, G., et al., *Endoglin expression is associated with poor oncologic outcome in oral and oropharyngeal carcinoma.* Acta Otolaryngol, 2006. **126**(6): p. 633-9.

61. Takahashi, N., et al., *Association of serum endoglin with metastasis in patients with colorectal, breast, and other solid tumors, and suppressive effect of chemotherapy on the serum endoglin.* Clin Cancer Res, 2001. **7**(3): p. 524-32.

62. Shiozaki, K., et al., *Antiangiogenic chimeric anti-endoglin (CD105) antibody: pharmacokinetics and immunogenicity in nonhuman primates and effects of doxorubicin.* Cancer Immunol Immunother, 2006. **55**(2): p. 140-50.

63. Warrington, K., et al., *Functional role of CD105 in TGF-beta1 signalling in murine and human endothelial cells.* Anticancer Res, 2005. **25**(3B): p. 1851-64.

64. MacDonald, T.J., D. Aguilera, and C.M. Kramm, *Treatment of high-grade glioma in children and adolescents.* Neuro Oncol, 2011. **13**(10): p. 1049-58.

65. Macdonald, D.R., et al., *Response criteria for phase II studies of supratentorial malignant glioma.* J Clin Oncol, 1990. **8**(7): p. 1277-80.

66. Batchelor, T.T., et al., *AZD2171, a pan-VEGF receptor tyrosine kinase inhibitor, normalizes tumor vasculature and alleviates edema in glioblastoma patients.* Cancer Cell, 2007. **11**(1): p. 83-95.

67. Gerstner, E.R., et al., *Infiltrative patterns of glioblastoma spread detected via diffusion MRI after treatment with cediranib.* Neuro Oncol, 2010. **12**(5): p. 466-72.

20. APPENDICES

20.1. Appendix 1: National Cancer Institute (NCI) Common Terminology Criteria for Adverse Events (CTCAE)

The NCI CTCAE (Version 4.0) should be used to assess Adverse Events and may be reviewed on-line at the following NCI website:

http://ctep.cancer.gov/protocolDevelopment/electronic_applications/docs/ctcaev4.pdf

20.2. Appendix 2: Karnofsky performance scale

Status	Karnofsky
Normal, no complaints	100
Able to carry on normal activities. Minor signs or symptoms of disease	90
Normal activity with effort	80
Care for self. Unable to carry on normal activity or to do active work	70
Requires occasional assistance, but able to care for most of his needs	60
Requires considerable assistance and frequent medical care	50
Disabled. Requires special care and assistance	40
Severly disabled. Hospitalization indicated though death nonimminent	30
Very sick. Hospitalization necessary. Active supportive treatment necessary	20
Moribund	10
Dead	0

20.3. Appendix 3: New York Heart Association Functional Capacity Classification

Class I. Patients with cardiac disease but without resulting limitation of physical activity. Ordinary physical activity does not cause undue fatigue, palpitation, dyspnea or anginal pain.

Class II. Patients with cardiac disease resulting in slight limitation of physical activity. They are comfortable at rest. Ordinary physical activity results in fatigue, palpitation, dyspnea or anginal pain.

Class III. Patients with cardiac disease resulting in marked limitation of physical activity. They are comfortable at rest. Less than ordinary activity causes fatigue, palpitation, dyspnea or anginal pain.

Class IV. Patients with cardiac disease resulting in inability to carry on any physical activity without discomfort. Symptoms of heart failure or the anginal syndrome may be present even at rest. If any physical activity is undertaken, discomfort increases.

20.4. Appendix 4: Bevacizumab Package Insert

The FDA approved bevacizumab package insert should be referenced and may be reviewed on-line at the following website:

http://www.gene.com/gene/products/information/pdf/avastin-prescribing.pdf
