# Supplementary material for: Endoglin inhibitor TRC105 with or without bevacizumab for bevacizumab-refractory glioblastoma (ENDOT): a multicenter phase II trial
Source: Commun Med (Lond). 2023 Sep 8;3:120. doi: 10.1038/s43856-023-00347-0 (PMC10491825; doi:10.1038/s43856-023-00347-0)
Supplement: Supplementary file 2 — Reporting Summary [file 43856_2023_347_MOESM2_ESM.pdf]

nature portfolio

Corresponding author(s): Manmeet Ahluwalia, MD, MBA (COMMSMED-23-0119A)

Last updated by author(s): Jul 7, 2023

Reporting Summary

Nature Portfolio wishes to improve the reproducibility of the work that we publish. This form provides structure for consistency and transparency in reporting. For further information on Nature Portfolio policies, see our Editorial Policies and the Editorial Policy Checklist.

Statistics

For all statistical analyses, confirm that the following items are present in the figure legend, table legend, main text, or Methods section.

n/a	Confirmed	
☐	☒	The exact sample size (n) for each experimental group/condition, given as a discrete number and unit of measurement
☐	☒	A statement on whether measurements were taken from distinct samples or whether the same sample was measured repeatedly
☐	☒	The statistical test(s) used AND whether they are one- or two-sided<br>*Only common tests should be described solely by name; describe more complex techniques in the Methods section.*
☐	☒	A description of all covariates tested
☐	☒	A description of any assumptions or corrections, such as tests of normality and adjustment for multiple comparisons
☐	☒	A full description of the statistical parameters including central tendency (e.g. means) or other basic estimates (e.g. regression coefficient) AND variation (e.g. standard deviation) or associated estimates of uncertainty (e.g. confidence intervals)
☐	☒	For null hypothesis testing, the test statistic (e.g. F, t, r) with confidence intervals, effect sizes, degrees of freedom and P value noted<br>*Give P values as exact values whenever suitable.*
☒	☐	For Bayesian analysis, information on the choice of priors and Markov chain Monte Carlo settings
☒	☐	For hierarchical and complex designs, identification of the appropriate level for tests and full reporting of outcomes
☒	☐	Estimates of effect sizes (e.g. Cohen's d, Pearson's r), indicating how they were calculated

Our web collection on statistics for biologists contains articles on many of the points above.

Software and code

Policy information about availability of computer code

Data collection	Data collection was done prospectively in an electronic database at all the sites (which were in US). This data was used for the trial assessments and outcomes.
Data analysis	Standard data analytics software were used (SAS, SPSS, STATA).

For manuscripts utilizing custom algorithms or software that are central to the research but not yet described in published literature, software must be made available to editors and reviewers. We strongly encourage code deposition in a community repository (e.g. GitHub). See the Nature Portfolio guidelines for submitting code & software for further information.

Data

Policy information about availability of data

All manuscripts must include a data availability statement. This statement should provide the following information, where applicable:

- Accession codes, unique identifiers, or web links for publicly available datasets
- A description of any restrictions on data availability
- For clinical datasets or third party data, please ensure that the statement adheres to our policy

Individual patient data (IPD) generated during this trial may be made available upon reasonable request, particularly for IPD meta-analysis, to the corresponding author (manmeetA@baptisthealth.net) beginning at publication and ending 36 months following publication. Deidentified data is not being made available publicly

given the low number of patients in the study (N=22), the relatively low incidence of glioblastoma (compared to other systemic malignancies) and the novelty of the study medication, all of which together hinder maintenance of patient confidentiality.

Human research participants

Policy information about studies involving human research participants and Sex and Gender in Research.

Reporting on sex and gender	This was a multi-center, open label phase 2 clinical trial of a novel drug for recurrent glioblastoma. Amongst all the cancers afflicting humans, glioblastoma is a rare but highly aggressive one, with only a few patients (amongst the total pool of cancer patients) being eligible for the current trial. Therefore, the key consideration was ensuring the trial reached appropriate power to study primary endpoints. Therefore, sex and gender were not explicitly considered in the design of the trial. However, all efforts were made by study investigators to enroll patients of all sexes/genders into the trial without any prejudice or bias. In the current study, 27% (6/22) were female patients across four sites in the US. Primary and secondary study outcomes were not stratified by gender, since this was not a pre-specified analysis or part of protocol amendment. However, aggregate data regarding the female participation in the trial has been reported. Consent was not taken to publish individual patient data for this work.
Population characteristics	22 patients with recurrent GBM that had progressed after chemoradiation and bevacizumab were enrolled at four sites in the United States, with 6 receiving TRC105 10 mg/kg weekly, in 4-week cycles, as single-agent therapy (cohort 1) and 16 patients receiving bevacizumab plus TRC105 in the combination portion of the study (cohort 2).<br><br>Median age was 54.5 years (range 31-70). 27% (6/22) were female. 9% (2/22) were African-American, 5% (1/22) were Asian and the rest (86%) were white. Further characteristics are described in Table 1 of the work.
Recruitment	Eligible participants were identified and recruited by physician specialists at each of the site participating in this multi-center trial. These were generally neuro-oncologists and/or medical oncologists.
Ethics oversight	The study was conducted at four centers in the US: Cleveland Clinic, Ohio, Case Western Reserve University, the Ohio State University, and the University of Cincinnati after institutional review board approval.

Note that full information on the approval of the study protocol must also be provided in the manuscript.

Field-specific reporting

Please select the one below that is the best fit for your research. If you are not sure, read the appropriate sections before making your selection.

☒ Life sciences ☐ Behavioural & social sciences ☐ Ecological, evolutionary & environmental sciences

For a reference copy of the document with all sections, see nature.com/documents/nr-reporting-summary-flat.pdf

Life sciences study design

All studies must disclose on these points even when the disclosure is negative.

Sample size	A one-stage accrual design with an accrual goal of 14 evaluable patients was initially planned to be employed to test the hypothesis that TRC105 can double the historical median time to progression in this population (i.e., from 1.5 months to 3.0 months). Assuming time to progression follows an exponential distribution, accrual takes approximately six months, and there is >6 months of additional follow-up once accrual has been completed, there would be 80% power to detect the specified difference using a two-sided test with 10% type I error. However, the protocol was amended. Therefore, a one-stage accrual design with an accrual goal of 22 evaluable patients was to be employed to test the hypothesis that TRC105 + bevacizumab could increase the median OS from 4 months to 7 months. Assuming OS followed an exponential distribution and an estimated accrual of approximately 12 months with 12 months of additional follow-up once accrual was completed, there would be 81% power to detect the specified difference using a two-sided test with 10% type I error. The safety population included all patients who received at least a portion of one dose of the study drug, TRC105. The evaluable population for determination of response included all patients with a baseline and a follow-up radiographic assessment for response at designated time points (e.g., every eight weeks).
Data exclusions	No data was excluded in this clinical trial, and all data for the primary and secondary outcomes was reported. For reporting of PFS, only patients where this duration could be calculated were included.
Replication	This was a phase 2 clinical trial. Further studies including larger and phase 3 trials are far better suited to target replication.
Randomization	This was a Phase 2 trial of just 22 patients. The decision to not randomize was taken given that (1) this was an early phase trial, (2) further RCTs would be based upon the results of this trial, and (3) patient accrual in an RCT is more challenging in a trial investigating the utility of a clinically unproven IND-status medication in a relatively rare tumor.
Blinding	This was an open-label trial. The decision to not have investigator-blinding and patient-blinding was taken to ensure enrollment in the trial, which was testing the utility of a clinically unproven IND-status medication in a relatively rare tumor.

Reporting for specific materials, systems and methods

We require information from authors about some types of materials, experimental systems and methods used in many studies. Here, indicate whether each material, system or method listed is relevant to your study. If you are not sure if a list item applies to your research, read the appropriate section before selecting a response.

Materials & experimental systems

n/a	Involved in the study
☐	☒ Antibodies
☒	☐ Eukaryotic cell lines
☒	☐ Palaeontology and archaeology
☒	☐ Animals and other organisms
☐	☒ Clinical data
☒	☐ Dual use research of concern

Methods

n/a	Involved in the study
☒	☐ ChIP-seq
☒	☐ Flow cytometry
☐	☒ MRI-based neuroimaging

Antibodies

Antibodies used	TRC105 (TRACON Pharmaceuticals, Inc., San Diego, CA) is a chimeric IgG1 antibody that binds human endoglin.
Validation	Manufactured by TRACON pharmaceuticals and supplied to trial sites as part of US FDA investigational new drug (IND) status.

Clinical data

Policy information about clinical studies

All manuscripts should comply with the ICMJE guidelines for publication of clinical research and a completed CONSORT checklist must be included with all submissions.

Clinical trial registration	NCT01564914
Study protocol	Protocol is available publicly - https://www.clinicaltrials.gov/ct2/show/NCT01564914
Data collection	Data collected from enrolled patients as part of clinical trial in an electronic database. Data was collected at four centers in the US: Cleveland Clinic, Ohio, the Case Western Reserve University, the Ohio State University, and the University of Cincinnati.
Outcomes	The primary endpoint in the initially planned study of single-agent TRC105 in rGBM was initially specified to be time to progression (TTP) for the tumor, defined as the time from consent to time of progression by RANO criteria. In light of suboptimal response rates in the single-agent cohort, the study protocol was amended to include another cohort of recurrent GBM given TRC105 combined with bevacizumab, and the primary endpoint was designated as overall survival (OS).<br><br>Other secondary endpoints included safety and tolerability, as assessed by the NCI Common Terminology Criteria for Adverse Events (CTCAE) version 4.0 (the version in effect when the trial started accruing), the overall response rate (ORR) as assessed by modified RANO criteria, progression-free survival (PFS), and PFS rate at six months (PFS-6).<br><br>Although the protocol specified TTP as an endpoint, PFS was also calculated given the US FDA guidance that PFS is preferred to TTP when the cause of death is likely due to cancer progression, as is the case of GBM. PFS was calculated from the time of consent till either death or progression as per RANO criteria. Median PFS and OS were visualized through Kaplan-Meier survival curves. Tumor responses and progression were evaluated using MRI per RANO criteria. Efficacy evaluations were performed at eight weeks intervals or earlier if disease progression was suspected.

Magnetic resonance imaging

Experimental design

Design type	Brain MRI done as part of a clinical trial.
Design specifications	Brain MRI done as part of a clinical trial.
Behavioral performance measures	Brain MRI done as part of a clinical trial.

Acquisition

Imaging type(s)	Brain MRI done as part of a clinical trial.
Field strength	As the study was a multi-center clinical trial of a drug for GBM, hence MRI was done for patients across institutions based on their imaging protocols (some with 1.5T and some with 3.0T). Brain MRI was done as part of a clinical trial.
Sequence & imaging parameters	As the study was a multi-center clinical trial of a drug for GBM, hence MRI was done for patients both using the center-specific MRI machines and at outside institutions as part of routine care (sequence and imaging parameters varied).
Area of acquisition	Whole Brain Scan as part of routine clinical care.

Diffusion MRI ☐ Used ☒ Not used

Preprocessing

Preprocessing software	MRI done for clinical care as part of standard of care practice for patients at the 4 different trial sites, each of which used their standard institutional procedures.
Normalization	MRI done for clinical care as part of standard of care practice for patients at the 4 different trial sites, each of which used their standard institutional procedures.
Normalization template	MRI done for clinical care as part of standard of care practice for patients at the 4 different trial sites, each of which used their standard institutional procedures.
Noise and artifact removal	MRI done for clinical care as part of standard of care practice for patients at the 4 different trial sites, each of which used their standard institutional procedures.
Volume censoring	MRI done for clinical care as part of standard of care practice for patients at the 4 different trial sites, each of which used their standard institutional procedures.

Statistical modeling & inference

Model type and settings	Brain MRI done as part of a clinical trial.
Effect(s) tested	Brain MRI done as part of a clinical trial.

Specify type of analysis: ☒ Whole brain ☐ ROI-based ☐ Both

Statistic type for inference (See Eklund et al. 2016)	N/A
Correction	N/A

Models & analysis

n/a	Involved in the study
☒	☐ Functional and/or effective connectivity
☒	☐ Graph analysis
☒	☐ Multivariate modeling or predictive analysis
